# Redox tuning of the H-cluster by second coordination sphere amino acids in the sensory [FeFe] hydrogenase from *Thermotoga maritima*†

**DOI:** 10.1039/d2sc06432d

**Published:** 2023-02-27

**Authors:** Nipa Chongdar, Patricia Rodríguez-Maciá, Edward J. Reijerse, Wolfgang Lubitz, Hideaki Ogata, James A. Birrell

**Affiliations:** a Max Planck Institute for Chemical Energy Conversion Stiftstraße 34-36 45470 Mülheim an der Ruhr Germany chongdar.nipa@gmail.com; b CSIR-National Institute of Oceanography Dona Paula-403004 Goa India; c Department of Chemistry, Inorganic Chemistry Laboratory, University of Oxford South Parks Road Oxford OX1 3QR UK; d Graduate School of Life Science, University of Hyogo Koto 3-2-1, Kamigori, Ako 678-1297 Hyogo Japan ogata@sci.u-hyogo.ac.jp; e School of Life Sciences, University of Essex Colchester CO4 3SQ UK james.birrell@essex.ac.uk

## Abstract

[FeFe] hydrogenases are exceptionally active catalysts for the interconversion of molecular hydrogen with protons and electrons. Their active site, the H-cluster, is composed of a [4Fe–4S] cluster covalently linked to a unique [2Fe] subcluster. These enzymes have been extensively studied to understand how the protein environment tunes the properties of the Fe ions for efficient catalysis. The sensory [FeFe] hydrogenase (HydS) from *Thermotoga maritima* has low activity and displays a very positive redox potential for the [2Fe] subcluster compared to that of the highly active prototypical enzymes. Using site directed mutagenesis, we investigate how second coordination sphere interactions of the protein environment with the H-cluster in HydS influence the catalytic, spectroscopic and redox properties of the H-cluster. In particular, mutation of the non-conserved serine 267, situated between the [4Fe–4S] and [2Fe] subclusters, to methionine (conserved in prototypical catalytic enzymes) gave a dramatic decrease in activity. Infra-red (IR) spectroelectrochemistry revealed a 50 mV lower redox potential for the [4Fe–4S] subcluster in the S267M variant. We speculate that this serine forms a hydrogen bond to the [4Fe–4S] subcluster, increasing its redox potential. These results demonstrate the importance of the secondary coordination sphere in tuning the catalytic properties of the H-cluster in [FeFe] hydrogenases and reveal a particularly important role for amino acids interacting with the [4Fe–4S] subcluster.

## Abstract 2

Site-directed mutagenesis of the sensory [FeFe] hydrogenase from *Thermotoga maritima* reveals new insight into how the protein environment tunes the active site properties for its sensory role.

## Graphical abstract

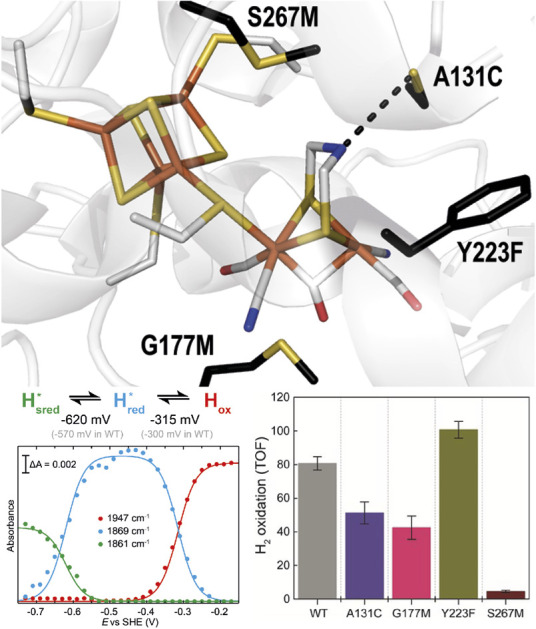

## Introduction

**Fig. 1:**
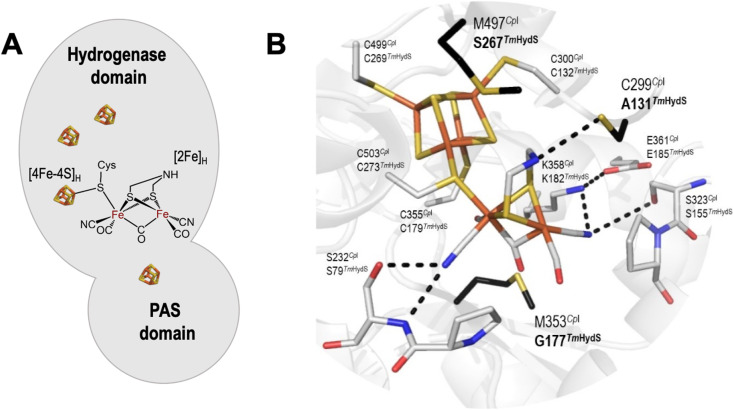
(A) Arrangement of cofactors in *Tm*HydS. The [4Fe–4S] clusters are shown as brown and yellow cubes and the [2Fe]_H_ cluster is shown in chemical structure format. (B) Close-up structural view of the H-cluster including the surrounding second coordination sphere amino acids. The structure is of the *C. pasteurianum* (*Cp*I) enzyme (PDB ID: 4XDC).^12^ Amino acid positions are labelled each with their *Cp*I (top) and *Tm*HydS (bottom) identities. Amino acids coloured in black (bold) were mutated in this study. The figure was prepared in Pymol. The dashed lines indicate hydrogen bonds between the H-cluster and surrounding amino acids. For an alternative view showing the location of Y223 see Fig. S2A.†

[FeFe]-hydrogenases catalyze the reversible conversion of protons and electrons to H_2_ at very high rates under ambient conditions and with minimal energy waste (overpotential).^1^ The active center of [FeFe] hydrogenases, known as the H-cluster, consists of a classical [4Fe–4S] cluster (called [4Fe–4S]_H_) covalently linked *via* a cysteine residue to a unique [2Fe] cluster (called [2Fe]_H_).^2^ In the latter, the iron atoms are coordinated by two terminal CO and CN^−^ ligands, a bridging CO ligand, and a bridging 2-azapropane-1,3-dithiolate (ADT) ligand (Fig. 1A).^3^ Based on amino acid sequences, phylogeny, subunit architectures and genetic organization, [FeFe] hydrogenases are classified into three groups: (a) prototypical and electron-bifurcating, (b) ancestral, and (c) sensory.^4^ Since the prototypical [FeFe] hydrogenases display excellent 2H^+^/H_2_ interconversion activity, this group has been most extensively studied.^5^ However, understanding other classes of [FeFe] hydrogenases is crucial for the general understanding of these fascinating enzymes. This information will unravel new facets that, in turn, can be beneficial for biomimetic research to develop affordable yet efficient hydrogen evolution catalysts. In an effort to look beyond the prototypical [FeFe] hydrogenase, we recently investigated the functional and spectroscopic properties of the sensory [FeFe] hydrogenase from *Thermotoga* (*T.*) *maritima* (*Tm*HydS).^6^

The genome of the anaerobic hyperthermophilic bacterium *T. maritima* contains genes encoding three different types of [FeFe] hydrogenases; an electron bifurcating hydrogenase, a prototypical hydrogenase and a sensory hydrogenase (HydS).^7,8^ Although the physiological role of HydS is unclear, it is assumed to regulate the expression of another hydrogenase gene present in the same operon, in response to changes in H_2_ concentration in the cell.^4,9^ In contrast to the other two groups of [FeFe] hydrogenases, HydS contains a PAS (Per–Arnt–Sim) domain along with the hydrogenase domain (Fig. 1A). PAS domains are versatile sensory modules found in signal transduction proteins from all three kingdoms of life. Despite having very low sequence conservation, PAS domains have a conserved three-dimensional scaffold consisting of a core with five antiparallel β-sheets and several flanking α-helices.^10,11^ When the PAS domain detects a physical or chemical stimulus, structural changes occur within the β-sheet core and the signal then propagates through the α-helices to the effector domain.^11^ The predicted regulatory function of HydS is based on the presence of the PAS domain.^9,13,14^

Sequence analysis of the protein scaffold surrounding the H-cluster, and comparison with known structures, revealed further deviating features of HydS compared to the prototypical [FeFe] hydrogenases.^6^ The amino acids in the polypeptide chain encompassing the H-cluster in prototypical [FeFe] hydrogenase are highly conserved. Mutating them has severe effects on catalytic activities of the protein, thus, indicating the importance of the protein framework in maintaining the enzyme's outstanding activity.^15^

Comparative sequence analyses of prototypical (*Clostridium pasteurianum* HydA1, *Cp*HydA1 or *Cp*I, for which the structure is known) and sensory (*Tm*HydS) [FeFe] hydrogenases showed that the cysteine residues ligating the iron atoms of the H-cluster and the residues stabilizing the proper conformation of the [2Fe] cofactor by H-bonding interactions are conserved in sensory type [FeFe] hydrogenases (Fig. 1B).^4,6,16^ However, three non-coordinating amino acids, C299, M353 and M497 (in *Cp*HydA1), which are also quite well conserved in prototypical [FeFe] hydrogenases, are altered in sensory type enzymes (Fig. 1B). Mutations of these residues, dramatically reduced the catalytic activity of these prototypical enzymes.^15^ Therefore, variations in amino acid identities surrounding the H-cluster in sensory and prototypical type [FeFe] hydrogenases could be the reason for their differences in catalytic activities and physiological function.

Recent work on a related sensory [FeFe] hydrogenase (*Tam*HydS) from *Thermoanaerobacter mathranii* shows high affinity for H_2_ as well as unusual reactivity with inhibitors.^16^ Furthermore, electrochemistry revealed irreversible behaviour not observed in the prototypical [FeFe] hydrogenases, which may however be related to its sensory function.^17^

In this work, we explored the role of those amino acids surrounding the H-cluster that show altered identities in sensory [FeFe] hydrogenases using site-directed mutagenesis. The resulting *Tm*HydS variants were biochemically and spectroscopically characterized in order to understand the role these amino acids play in regulating catalytic activity and the electronic structure of the H-cluster. In particular, we found that mutating S267 (equivalent to M497 in *Cp*HydA1) to methionine resulted in a substantial loss in activity, which appears to result from a change in the redox potential of the [4Fe–4S]_H_ subcluster. The effects on the catalytic activity and the differences with the prototypical hydrogenase are discussed.

## Results

### Selection of amino acid residues for mutation

Crystal structure analysis of *Cp*I and *Dd*HydAB (*Dd*H) [FeFe] hydrogenases, together with theoretical calculations (quantum mechanics/molecular mechanics and molecular dynamics) on the same structures, suggested that the highly conserved residue C299 (*Cp*I), present in the vicinity of the amine bridge-head of the ADT ligand, serves as the immediate proton acceptor/donor for the H-cluster in prototypical [FeFe] hydrogenases.^1,18–23^ As expected, substituting this cysteine in any prototypical [FeFe] hydrogenase to other amino acids, except aspartic acid, resulted in complete loss or severe impairment of the catalytic activity.^15,19^ In sensory [FeFe] hydrogenases, this position is occupied by a cysteine residue only in 17% of sequences (taken from the HydDB database of Søndergaard *et al.*^24^), while other sequences contain residues like alanine (≈43%), proline (≈10%), serine (≈10%), *etc.* (Fig. S1†). These residues, especially alanine, do not appear to be suitable proton acceptor/donor sites, thus, suggesting that the proton channel may be different in sensory [FeFe] hydrogenases. Therefore, such non-conservative variations in amino acid identity suggest that this position may not play an important role in the functioning of HydS. Despite the fact that *Tm*HydS contains alanine in this position (A131) it shows much higher activity than the C299A variant of *Cp*I (or the C169A variant of *Cr*HydA1).^15,20^ On the other hand, the activity of *Tm*HydS is much lower than WT *Cp*I and *Cr*HydA1. In an attempt to increase the activity of *Tm*HydS, and further study the role of this amino acid, we mutated residue A131 in *Tm*HydS to cysteine.

Another residue in prototypical [FeFe] hydrogenases that interacts with the bridge-head amine group of the ADT ligand is a conserved methionine (M497 in *Cp*I), whose mutation to leucine severely reduced the catalytic activity of the enzyme.^15^ Spectroscopic analysis indicated that the M497L variant lacked a properly integrated [2Fe]_H_ cluster, explaining its diminished activity.^15^ In sensory [FeFe] hydrogenases, however, the amino acid in this position is not conserved (Fig. S1†). In *Tm*HydS, this position (267) is occupied by a serine residue (Fig. 1B). In spite of this, the artificially maturated protein showed typical spectroscopic signatures, indicating proper incorporation of the [2Fe]_H_ cluster in *Tm*HydS.^6^ Here, we have mutated this residue to methionine (S267M variant) to explore the function of this position in *Tm*HydS.

The third residue, a conserved methionine in prototypical [FeFe] hydrogenases (M353 in *Cp*I), has been proposed to stabilize the bridging CO ligand through electrostatic interaction between the sulfur atom of the methionine and the oxygen atom of the bridging CO. Mutating M353 in prototypical [FeFe] hydrogenases also lowered their catalytic activity, but not as severely as the other two aforementioned residues.^15^ In sensory [FeFe] hydrogenases, this position is mostly occupied by small amino acids like glycine (≈51%), serine (≈32%) or threonine (≈17%). In the prototypical [FeFe] hydrogenases *Cp*II and *Cp*III this residue is replaced by Thr and Gly, respectively.^25–28^ These enzymes show high levels of activity, however, with a strongly altered catalytic bias. In *Tm*HydS, this position is occupied by a glycine (G177) and we have also mutated this residue in *Tm*HydS (G177M variant) to get some insight on how this position influences the properties of the H-cluster in sensory [FeFe] hydrogenases.

From a homology modelled structure of *Tm*HydS, we hypothesize that Y223 (corresponds to the F417 residue in *Cp*I), which lies in the vicinity of the amine-bridge of the H-cluster, could act as an alternative proton transfer residue (Fig. S2A†). It is notable here that in prototypical [FeFe] hydrogenases, this position is occupied by a phenylalanine residue (Fig. S2B†). Therefore, apart from the three aforementioned amino acids, residue Y223 in *Tm*HydS was mutated to phenylalanine to investigate this hypothesis.

### Isolation of the apo *Tm*HydS variants

The *Tm*HydS variants, A131C, S267M, G177M and Y223F were produced by site directed mutagenesis using the primers listed in Table S1† and the plasmid of WT-*Tm*HydS as a template (for the detailed method, please see ESI text 1†). The apo-forms (protein containing only the four [4Fe–4S] clusters but lacking the [2Fe] subsite of the H-cluster) of the *Tm*HydS variants were prepared using our previously published protocol.^6^ The yields of all variants except G177M (which decreased by ≈50%, data not shown) were comparable to WT-*Tm*HydS.

Like WT-*Tm*HydS, the UV-vis spectra of all variants showed a broad feature in the 350–500 nm region with the highest intensity at 410 nm, indicating the presence of [4Fe–4S] clusters (Fig. S3†). The ratio of the absorption intensities at 280 nm and 400 nm, which provide a qualitative measure of FeS cluster incorporation, were for all variants similar to that of the wild type, indicating that the FeS cluster assembly was not affected by the mutations. Since a fully assembled [4Fe–4S]_H_ cluster is a prerequisite for the successful incorporation of the [2Fe]^ADT^ complex,^29^ we determined the iron content of the *Tm*HydS variants to estimate the number of FeS clusters in the proteins (Table S2†). Like WT-*Tm*HydS, all variants possessed ≈15 moles iron per mole of protein, in agreement with the expected presence of four [4Fe–4S] clusters. To obtain the holo-form of the *Tm*HydS variants, the purified apo-proteins were incubated with [2Fe]^ADT^ complex (see methods in ESI, ESI text 1†), and after removal of the excess [2Fe]^ADT^, the spectroscopic properties and the enzymatic activities of these variants were examined in detail.

### Spectroscopic characterization of the *Tm*HydS variants

In order to determine whether the *Tm*HydS variants were successfully maturated using the ‘artificial-maturation’ technique, Fourier transform infrared (FTIR) spectroscopy was performed on these proteins. Compared to the [2Fe]^ADT^ complex in solution, the ‘as-isolated’ samples of the *Tm*HydS variants showed significantly narrower CO and CN^−^ FTIR bands, indicating successful incorporation of the [2Fe]_H_ cofactor (Fig. S4A†). In the as-isolated state, the variants A131C, G177M and Y223F showed FTIR bands at very similar positions (2055, 2022, 1893, 1872 and 1762 cm^−1^) to those of WT-*Tm*HydS, whereas, the S267M variant showed small (2–4 cm^−1^) red-shifts (*i.e.* lower wavenumbers) of all FTIR bands (except the bridging CO band) (Fig. S4A†). This suggests that none of these mutations led to major changes in the interactions between the polypeptide chain forming the active site pocket and the H-cluster. Additionally, the presence of just five IR bands with similar energies suggests that all variants exist in a single redox state under these conditions (2% H_2_, pH 8, 15 °C), as observed previously for the WT enzyme.^6^ In the WT enzyme this state was determined to be the one-electron reduced state (named 
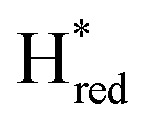
, *vide infra*). The fact that all the variants appear to be in the same state under these conditions suggests that the electronic structure of the H-cluster has not been significantly perturbed.

Next, we determined if all *Tm*HydS variants have similar levels of H-cluster occupancy by comparing the ratios of intensity of the most intense CO stretching band (1872 cm^−1^) to the intensity of the amide II band at 1540 cm^−1^ (Fig. S4B†). In the A131C, Y223F and S267M variants this ratio was found to be comparable to WT-*Tm*HydS indicating that the incorporation of the [2Fe]_H_ cluster is not affected by these mutations. In the case of the G177M variant, this ratio was approximately 2-fold lower than WT-*Tm*HydS (Fig. S4C†). It is possible that the bulky methionine side chain disturbs the integration of the [2Fe]_H_ subsite.

**Fig. 2:**
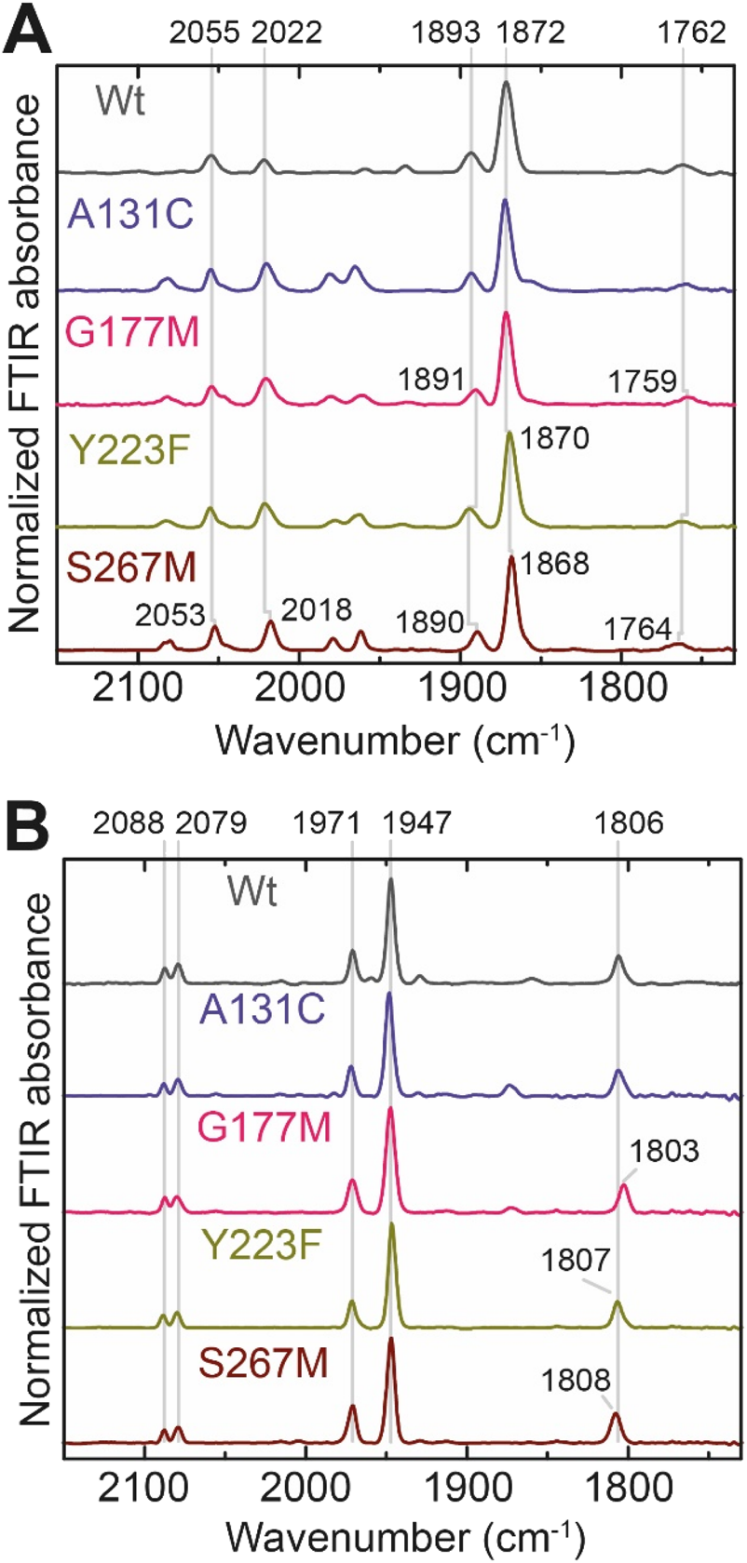
(A) FTIR spectra of *Tm*HydS variants reduced with sodium dithionite (≈10 mM) at pH 8 (50 mM Tris, 200 mM KCl) under 2% H_2_. The additional peaks between 1950 and 2000 cm^−1^ could represent the H_hyd:red_ or H_hyd:ox_ states with an over-oxidized [2Fe]_H_ subcluster and a reduced or oxidized [4Fe–4S]_H_ subcluster, respectively. (B) FTIR spectra of *Tm*HydS variants oxidized with thionine or hexaammineruthenium(iii) chloride (S267M) at pH 8 under 100% N_2_.

The positions of CO and CN^−^ vibrational bands in FTIR spectroscopy serve as excellent indicators of the redox-state of the H-cluster. This is because reduction of the H-cluster leads to increased electron density on the Fe ions, which increases backbonding into the CO and CN^−^ ligand π* orbitals, which weakens and lengthens the CO and CN^−^ bonds and shifts their IR vibrational frequencies to lower energy.^5^ Formal reduction of the [2Fe]_H_ subcluster gives the largest shifts (≈50 cm^−1^), while formal reduction of the [4Fe–4S]_H_ subcluster only partially increases the electron density on [2Fe]_H_, giving smaller shifts (≈10 cm^−1^). Hence, this method is widely used in [FeFe] hydrogenase research. We have previously shown that the as-isolated state in WT-*Tm*HydS is a one-electron reduced state in which the electron density mostly resides on [2Fe]_H_, the so-called (unprotonated) 
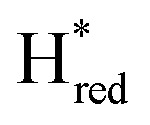
 state [4Fe–4S]^2+^–[Fe(i)–Fe(i)] in which electron and proton transfer are decoupled.^6^ As mentioned before, all four variants showed the same FTIR bands in the as isolated state. Therefore, it appears that all four variants are in the 
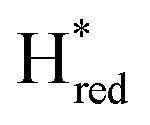
 state under these conditions. Treatment of all four variants with a reducing agent (sodium dithionite) resulted in FTIR spectra (Fig. 2A) essentially the same as the as-isolated state, confirming that the active center is already reduced. In the A131C variant, a shoulder at 1860 cm^−1^ is visible, probably from partial further reduction to the 
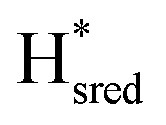
 state [4Fe–4S]^+^–[Fe(i)–Fe(i)], where [4Fe–4S]_H_ is also reduced. This may indicate a slightly more positive redox potential for the 
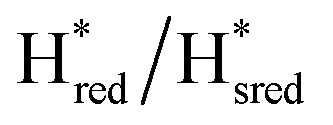
 state in this variant. Moreover, peaks at 1960 cm^−1^ and 1980 cm^−1^ are visible, possibly from an additional, as yet unidentified, reduced state of *Tm*HydS. Evidence for this state is also present in the other variants at similar energies. Based on similarities of the positions of the FTIR bands, this could represent the H_hyd:red_ or H_hyd:ox_ states with an over-oxidized [2Fe]_H_ subcluster and a reduced or oxidized [4Fe–4S]_H_ subcluster, respectively.^30^

We then oxidized the variants using the redox dye, thionine. The FTIR spectra of A131C, Y223F and G177M variants showed large (>35 cm^−1^) blue-shifts of the CO and CN^−^ ligand vibrational bands following treatment with thionine, suggesting oxidation of [2Fe]_H_ to give the most oxidized state, the so-called H_ox_ state [4Fe–2S]^2+^–[Fe(ii)–Fe(i)]. However, thionine treatment was insufficient to oxidize the S267M variant and so a slightly stronger oxidizing agent, hexaammineruthenium(iii) chloride (HAR) was used (Fig. 2B). The FTIR peaks of thionine- or HAR-oxidized samples of the *Tm*HydS variants appeared at similar positions (Fig. 2B) and resembled the FTIR spectra of the H_ox_ state from WT-*Tm*HydS. In the A131C variant, a residual peak from the 
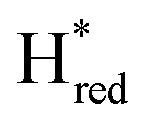
 state in the thionine-oxidized sample can still be observed.

**Fig. 3:**
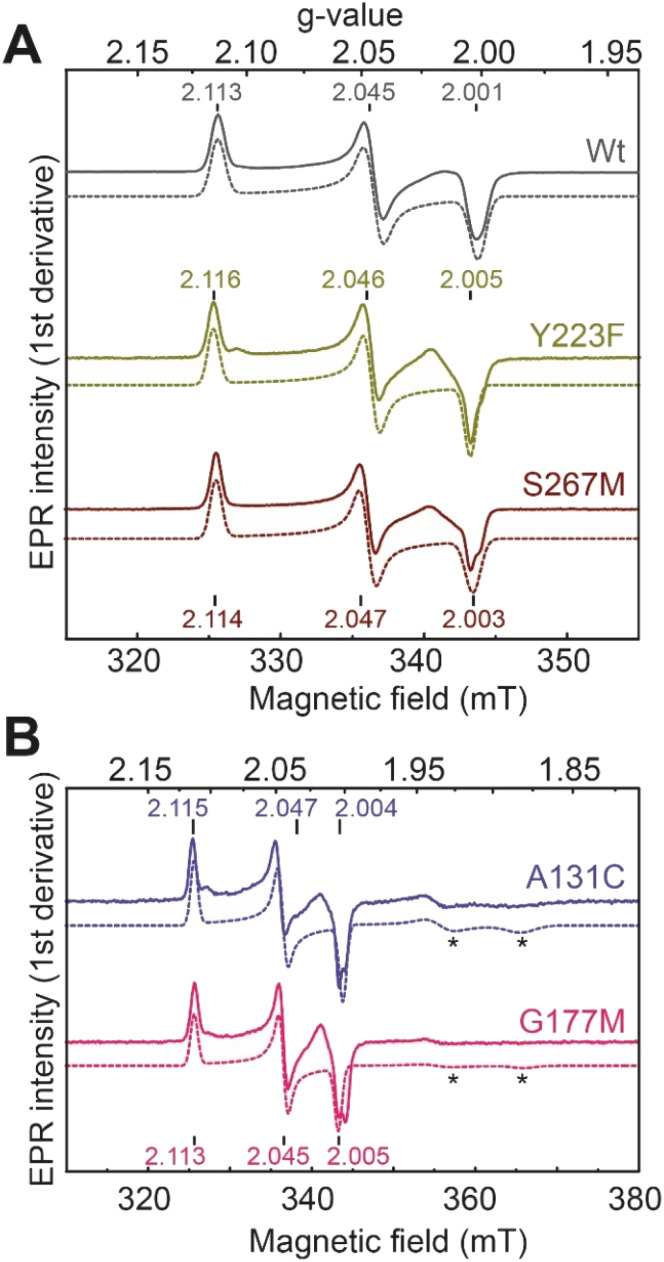
CW X-band (≈9.64 GHz) EPR spectra of *Tm*HydS variants (A) wild type, Y223F and S267M, and (B) A131C and G177M, oxidized with thionine or hexaamineruthenium(iii) chloride (for S267M) at pH 8 under 100% N_2_. Experimental spectra are shown in solid lines and simulations are shown as dashed lines. Evidence for the thionine radical can be identified from perturbations around the *g* = 2 region. The spectra were measured at 20 K, with 10 μW microwave power using 7.46 Gauss modulation amplitude. All other parameters are described in the methods section (see ESI†). The asterisks (*) indicate contributions from [4Fe–4S]^+^ clusters.

We next performed electron paramagnetic resonance (EPR) spectroscopy on all these oxidized proteins to analyze how the mutations affect the magnetic properties of the H-cluster. In the H_ox_ state, [2Fe]_H_ is in a mixed valent Fe(ii)Fe(i) configuration and [4Fe–4S]_H_ is in the 2+ state, giving overall an EPR active, *S* = ½, state with a characteristic rhombic EPR spectrum. Apart from small differences around the *g* = 2 region attributable to the reduced thionine radical, the EPR spectra of all the variants are essentially identical but with small shifts in the *g*-values of the features in the spectrum, which can be assigned to small perturbations in the electronic structure of the H-cluster (Fig. 3).

### Catalytic activity of the *Tm*HydS variants

**Fig. 4:**
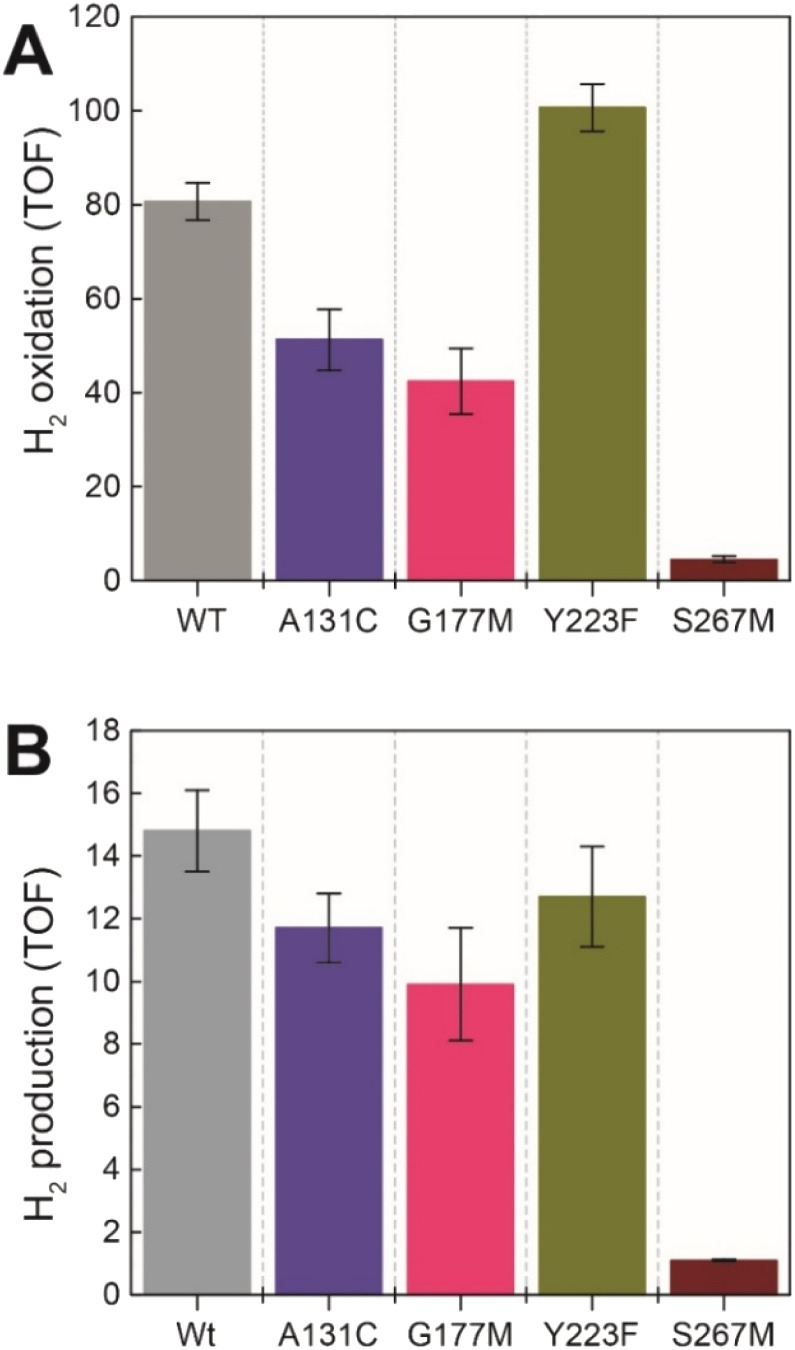
(A) H_2_ oxidation activity (s^−1^) for the *Tm*HydS variants measured at 70 °C in 200 mM phosphate buffer pH 8 under a 100% H_2_ atmosphere following 1 mM benzyl viologen reduction using UV-vis absorbance at 600 nm. (B) H_2_ production activity (s^−1^) measured at 70 °C with gas chromatography after 10 min of incubation with 100 mM sodium dithionite and 10 mM methyl viologen.

Next, we assessed the enzyme activity of the variants using viologen based H_2_ oxidation and H^+^ reduction solution assays at 70 °C, the optimal temperature for enzyme activity in WT-*Tm*HydS (for experimental details see ESI†). In comparison to WT-*Tm*HydS, the A131C variant showed a 40% decrease in H_2_ oxidation activity (Fig. 4A and B) and a 20% decrease in H_2_ production activity. Initially, we had expected that incorporation of a cysteine at the end of the traditional proton transfer pathway to the H-cluster might restore proton transfer and, therefore, facilitate much higher activity. However, it is important to note that other amino acids within this pathway (E279, E282, S319 in *Cp*I) are also different in HydS and other sensory [FeFe] hydrogenases (Fig. S5†), and that studies on prototypical [FeFe] hydrogenases have shown each of these residues to play a critical role in proton transfer.^20^ Nevertheless, the results from this variant strongly imply that HydS has an alternative proton transfer pathway. Additionally, the decrease in activity of the A131C variant compared with WT suggests that the cysteine in this position interacts with the ADT ligand in the H-cluster in this variant, possibly interfering with the alternative proton transfer pathway.

The G177M variant showed 50% lower H_2_ oxidation and 30% lower proton reduction activities (Fig. 4). This result fits well with the lower loading of [2Fe]_H_ in this variant mentioned earlier, thus, on a per [2Fe]_H_ site basis the activity is very similar to WT. The Y223F variant showed somewhat higher H_2_ oxidation activity (≈25% higher) than the WT, and marginally lower (≈10% lower) H_2_ production activity compared to the WT, suggesting that this residue does not play a role in proton transfer in *Tm*HydS (Fig. 4).

Unexpectedly the S267M variant shows a drastic drop in both H_2_ oxidation (≈95% lower than WT) and H_2_ production (93% lower than WT) activities (Fig. 4). Such a severe effect on activity indicates that S267 plays a crucial role in *Tm*HydS and its mutation could have introduced some local structural deformation in the protein. However, this possibility can be ruled out based on the fact that S267M shows WT-like FTIR and EPR spectral features, which suggest that the conformation surrounding the H-cluster remains unaffected by this mutation. Another possibility that the S267M mutation could introduce a specific temperature-dependent instability can be excluded since H_2_ oxidation assays, which were initiated by adding *Tm*HydS stored at room temperature to the pre-warmed assay buffer in a temperature-controlled cuvette holder, showed stable kinetics over the course of the measurement (Fig. S6†). Intrigued by this result, we proceeded to inspect whether: (i) S267 is a possible proton transfer residue, and mutating it to methionine blocks the proton transfer pathway, or (ii) S267 is important for the regulation of the redox potential of the H-cluster and the mutation to methionine disturbs that potential control.

### Does S267 act as a proton transfer residue?

**Fig. 5:**
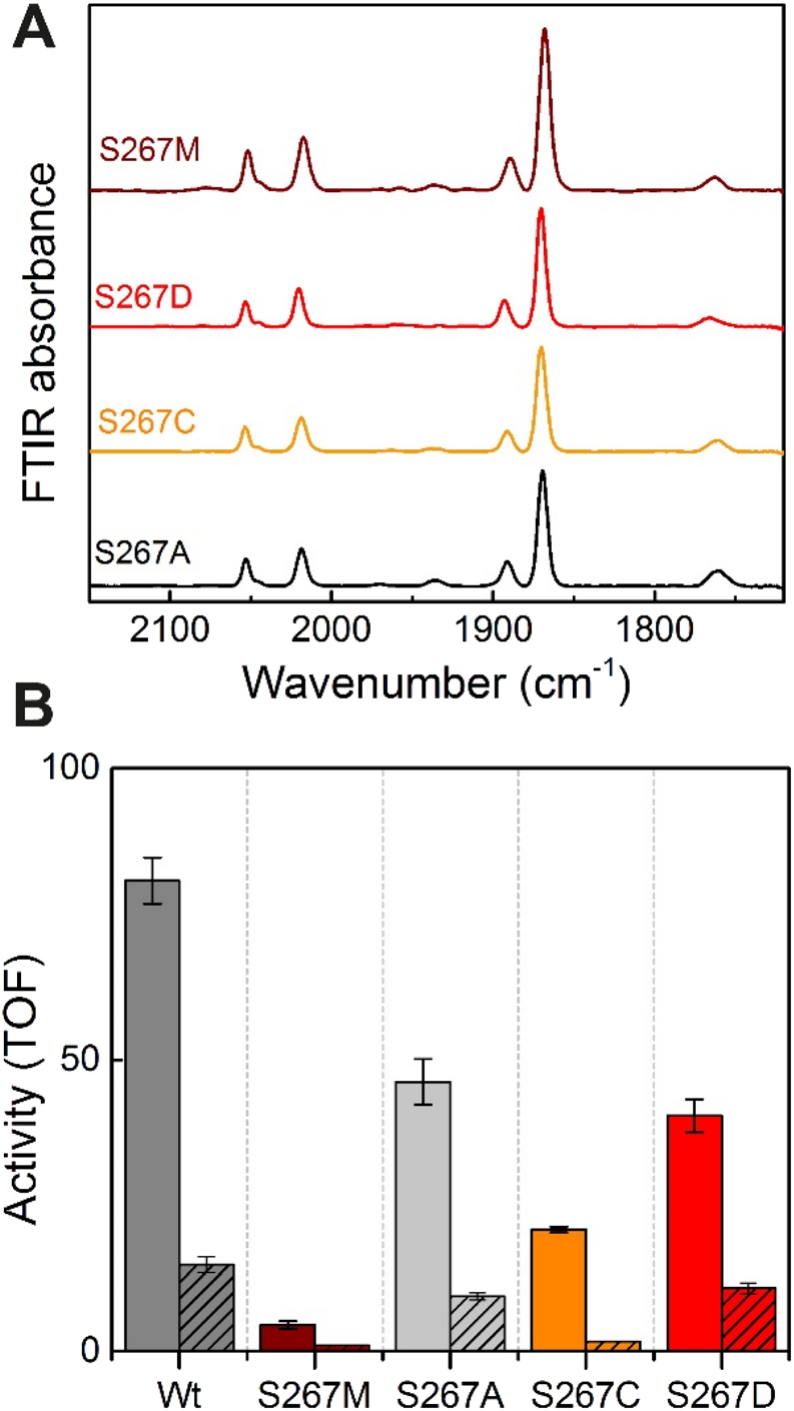
(A) FTIR spectra of *Tm*HydS S267 variants as isolated at pH 8 (50 mM Tris–HCl, 200 mM KCl) under 2% H_2_. (B) H_2_ oxidation (filled bars) and H^+^ reduction (striped bars) activity (s^−1^) for the *Tm*HydS S267 variants. H_2_ oxidation was measured at 70 °C in 200 mM phosphate buffer pH 8 under a 100% H_2_ atmosphere by following 1 mM benzyl viologen reduction using UV-vis absorbance at 600 nm. H^+^ reduction activity was measured at 70 °C with gas chromatography after 10 min of incubation with 100 mM sodium dithionite and 10 mM methyl viologen.

Proton-transfer is essential in many proteins, including hydrogenases.^31^ In the protein matrix, proton transfer is mediated either by polar or charged amino acids, or trapped water molecules. Serine is a small polar amino acid, which may participate in proton transfer, however, if mutated to the nonpolar amino acid methionine, this functionality would be lost. In order to check if the residue S267 is indeed involved in proton transfer, this position was further mutated to alanine, aspartic acid and cysteine, thus generating three additional variants S267A, S267D, and S267C. After, purification of the apo-protein, these variants were maturated with [2Fe]^ADT^ and their FTIR spectra were recorded. As, shown in Fig. 5, all the variants showed spectra similar to WT-*Tm*HydS and S267M. We anticipated that, if the hydroxyl side chain of serine is critical for proton transfer, the S267A mutant would show lower H_2_ conversion activity than WT, possibly as low as the S267M variant, while S267C and S267D may retain the ability to transfer protons and show activity comparable to WT-*Tm*HydS. However, as shown in Fig. 5, the H_2_ oxidation and production activities of the S267A variant are just ≈ 50% lower than that of the WT. The S267D variant shows comparable activity to S267A, and surprisingly the activity of the S267C variant is even lower (≈20% of WT). These observations question the role of S267 in the proton-transfer in *Tm*HydS. It is possible that the small CH_3_ side chain of S267A can accommodate a water molecule, which would compensate for the lack of polar groups.

### Does S267 influence the redox potential of the H-cluster?

The fact that the S267M variant could not be oxidized by thionine suggested that this mutation has influenced the redox potential of the H-cluster. In order to determine the redox potential of the H-cluster in this variant, IR spectroelectrochemical titrations were performed. This method is widely used to determine the potential dependence of the redox-states in hydrogenases, as they contain intrinsic IR active probes such as the CN^−^ and CO ligands in their active site, which are extremely sensitive to electron density changes within the H-cluster. IR spectroelectrochemical experiments were performed by mixing the holo-S267M variant with a redox-mediator cocktail, to ensure fast electron transfer and, therefore, shorter equilibrations times. Then, the sample was loaded into a home-made transmission FTIR cell, equipped with a three-electrode system for poising the potential at the desired values.^32^

**Fig. 6:**
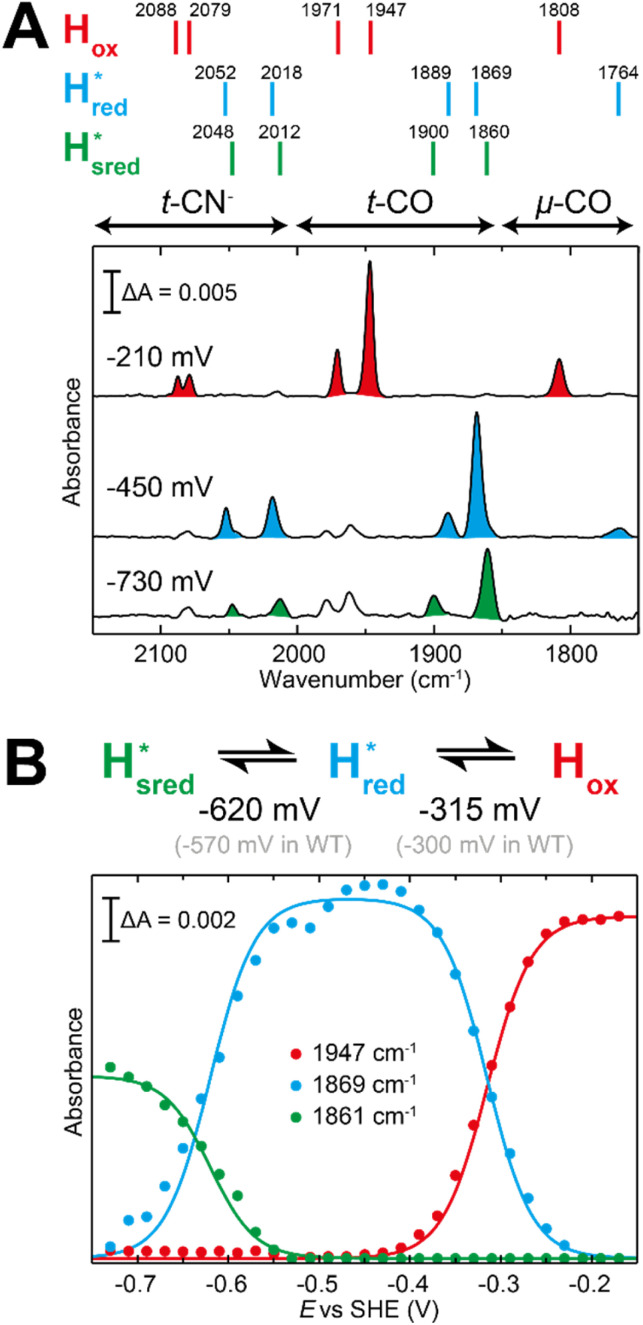
Reductive IR spectroelectrochemical titration of *Tm*HydS S267M. (A) FTIR spectra of *Tm*HydS S267M at various applied potentials (*vs.* SHE) showing typical spectra of the H_ox_ (red), 
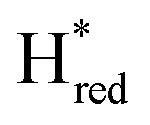
 (blue) and 
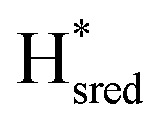
 (green) states. (B) The intensity of the most intense band in the H_ox_ (1947 cm^−1^), 
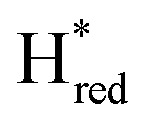
 (1869 cm^−1^) and 
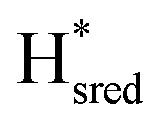
 (1861 cm^−1^) states, the latter two were determined from fitting Gaussian peaks (see Fig. S8†), is plotted against the applied potential and fitted with a model based on the Nernst equation using *n* = 1 for the 
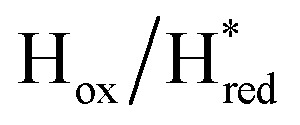
 (*E*_m_ = −315 mV) and 
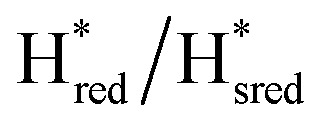
 (*E*_m_ = −620 mV) transitions.

The reductive titration of holo-S267M commenced at a mildly negative potential (−130 mV, *vs.* SHE), where the FTIR spectrum of the protein is indistinguishable from that of the HAR-oxidized sample, indicating that the H-cluster is in the H_ox_ state (Fig. 6). As the potential was lowered, the intensities of these FTIR bands decreased, while a new set of bands started to appear from the 
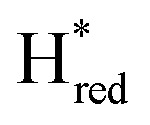
 state. After reaching the highest intensities, at ≈−450 mV *vs.* SHE, the intensities of these bands decreased as the potential was poised to even more negative values, and new bands appeared from the 
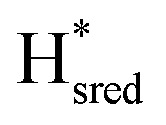
 state. The intensities of this new set of FTIR bands reached a plateau at ≈−700 mV *vs.* SHE. The oxidative titrations showed essentially the same behavior (Fig. S7†).

The intensity of the H_ox_ state was possible to extract directly by following the intensity at 1947 cm^−1^. However, due to the fact that the peaks from the 
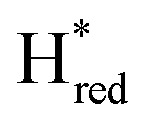
 and 
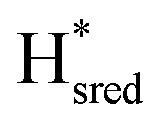
 states overlap, the intensities of the individual components were extracted by fitting the region between 1880 cm^−1^ and 1850 cm^−1^ with Gaussian peaks (Fig. S8†) with maxima at 1869 cm^−1^
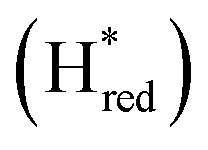
 and 1861 cm^−1^
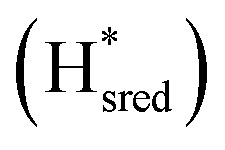
. The titration curves obtained by following the intensity of the most prominent FTIR band in each state could be fitted with the Nernst equation corresponding to two successive one-electron reductions, of which the first reduction occurs at ≈−315 mV *vs.* SHE, and the second at ≈−620 mV *vs.* SHE. The redox potential of the H_ox_ to 
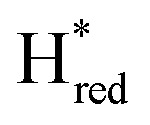
 transition in the S267M variant is similar to that of WT-*Tm*HydS (−300 mV *vs.* SHE)^6^ but the 
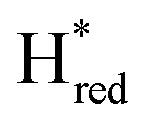
 to 
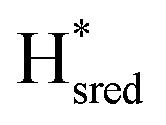
 transition is 50 mV more negative (−620 mV) than in WT (−570 mV, *vs.* SHE).^6^ The oxidative titrations gave very similar values for the two potentials (Fig. S7†). Since the 
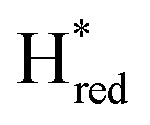
 to 
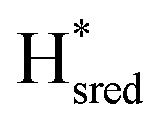
 state is thought to involve reduction of [4Fe–4S]_H_, we hypothesize that S267 interacts with [4Fe–4S]_H_, possibly through hydrogen bonding with its hydroxyl group, to stabilize the reduced state of the cluster. When this amino acid is mutated to Met in S267M, the amino acid can no longer engage in hydrogen bonding and the redox potential of the cluster becomes more negative, thus it is more difficult to reduce.

### Spectroelectrochemical characterization of S267M^PDT^ variant

Since the discovery of artificial maturation,^33,34^ [FeFe] hydrogenases were produced with several derivatives of the [2Fe]^ADT^ cofactor to investigate the importance of the chemical properties of the [2Fe]_H_ subcluster.^35–38^ Among these derivatives, the cofactor containing propane-1,3-dithiolate (PDT) instead of ADT, in which the amine head group of the cofactor is replaced with the non-protonatable methylene group, is used to investigate the role of proton coupled electron transfer in the H-cluster. In our previous study, spectroelectrochemical FTIR analysis of WT *Tm*HydS^PDT^ revealed a unique property of this enzyme, that the H-cluster does not need protonation of the bridging dithiolate ligand for reduction of [2Fe]_H_.^6^ This was illustrated by the fact that the one-electron reduced *Tm*HydS^PDT^ displayed the spectra of both the 
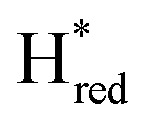
 (reduced [2Fe]_H_) and H_red_ (reduced [4Fe–4S]_H_) states, and could be further reduced to the 
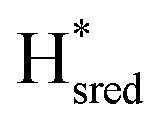
 state. This is in stark contrast to the PDT versions of prototypical hydrogenases, which only show the one-electron reduced H_red_ state, and cannot be reduced by two electrons.^39–41^ It should be noted that for WT *Tm*HydS, the reason why the H_red_ state is not observed in the ADT version of the enzyme is that the nitrogen of the ADT ligand is more electron withdrawing than the carbon of the PDT ligand, which gives [2Fe]^ADT^ a more positive redox potential than that of [2Fe]^PDT^.

**Fig. 7:**
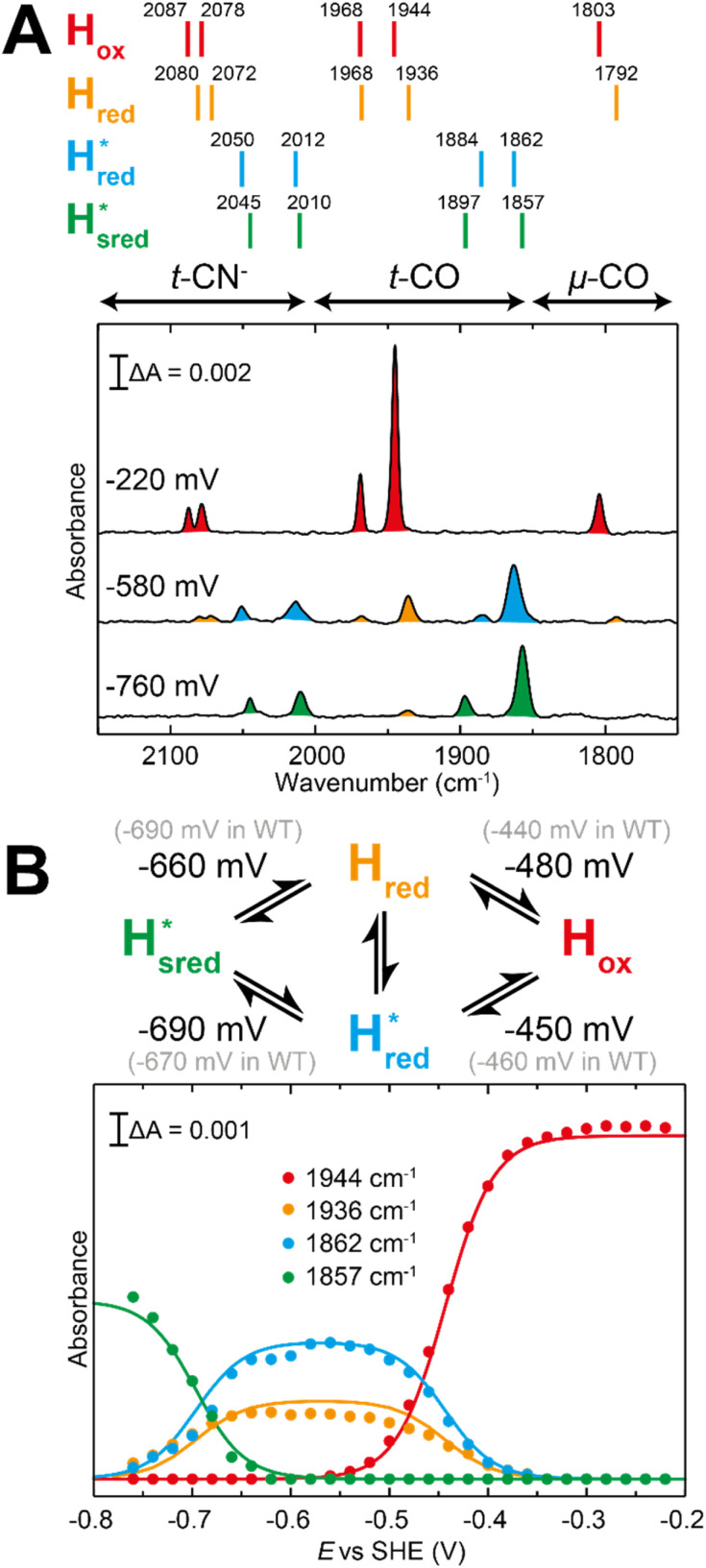
Reductive IR spectroelectrochemical titration of *Tm*HydS^PDT^ S267M. (A) FTIR spectra of *Tm*HydS^PDT^ S267M at various applied potentials (*vs.* SHE) showing typical spectra of the H_ox_ (red), H_red_ (orange), 
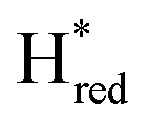
 (blue) and 
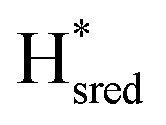
 (green) states. (B) The intensity of the most intense band in the H_ox_ (1944 cm^−1^), H_red_ (1936 cm^−1^), 
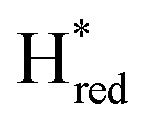
 (1862 cm^−1^) and 
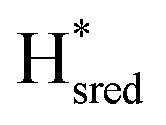
 (1857 cm^−1^) states, all determined from fitting Gaussian peaks (see Fig. S10†), is plotted against the applied potential and the data are fitted with a model based on the Nernst equation using *n* = 1 for the H_ox_/H_red_ (*E*_m_ = −480 mV), 
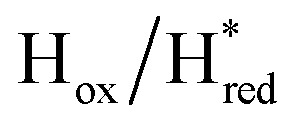
 (*E*_m_ = −450 mV), 
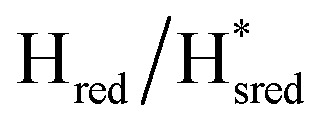
 (*E*_m_ = −660 mV) and 
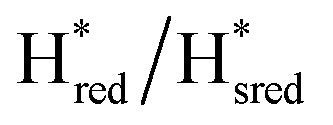
 (*E*_m_ = −690 mV) transitions.

In order to verify if the S267M variant also maintains this exceptional behavior, we performed an IR spectroelectrochemical titration on the PDT derivate of the S267M variant (S267M^PDT^). The reductive titration of this variant was carried out in the same way as explained above for S267M^ADT^. At oxidizing potentials, S267M^PDT^ showed FTIR bands similar to the H_ox_ state and, as the potential was decreased below −310 mV, the intensities of these peaks dropped and new peaks started to appear (Fig. 7). The number of FTIR peaks showed that there are at least two states appearing, similar to those observed for WT-*Tm*HydS^PDT^. The positions of one set of FTIR bands were shifted to lower energy relative to the H^PDT^_ox_ state by <10 cm^−1^, while the other set of peaks was shifted by >40 cm^−1^ to lower energy, indicating that the first set of bands belongs to a species where [4Fe–4S]_H_ is reduced (the H_red_ state) while the second species formed is due to the reduction of [2Fe]_H_ (the 
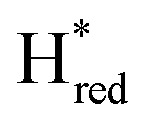
 state). In terms of intensities, these peaks reached their saturation at ≈−500 mV, and remained stable until ≈−600 mV, after which a new set of peaks started to appear, at positions similar to the 
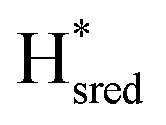
 state. The oxidative titration showed essentially the same behaviour (Fig. S9†).

Since there is substantial overlap of the peaks in the H_ox_ and H_red_ states as well as for the peaks in the 
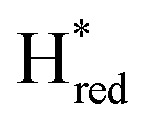
 and 
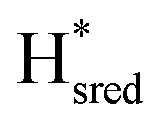
 states, Gaussian peak fitting was applied (Fig. S10†). The intensities of the individual components were extracted by fitting the region from 1955 cm^−1^ to 1925 cm^−1^ (for H_ox_/H_red_) and 1875 cm^−1^ to 1845 cm^−1^ (for 
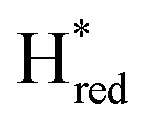
 and 
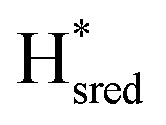
) using Gaussian peaks with maxima at 1944 cm^−1^ (H_ox_), 1936 cm^−1^ (H_red_), 1862 cm^−1^ (
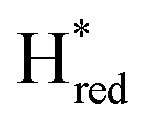
) and 1857 cm^−1^ (
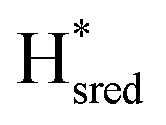
). The reductive titration curves can be fitted with the Nernst equation following the model shown in Fig. 7B. Overall, S267M^PDT^, behaved in a way similar to that of WT-*Tm*HydS^PDT^. However, the ratio of the H_red_ to 
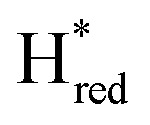
 states is altered such that 
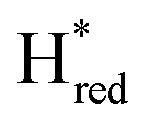
 dominates for S267M^PDT^, while H_red_ dominates for WT *Tm*HydS^PDT^. This indicates a decreased thermodynamic stability of the H_red_ state in comparison with the 
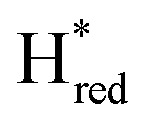
 state, in agreement with a more negative redox potential for [4Fe–4S]_H_ in S267M. Indeed, fitting of the data with the Nernst equation showed that the H_ox_ to 
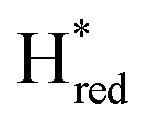
 transition has a redox potential (−450 mV) similar to WT (−460 mV), whereas the H_red_ to 
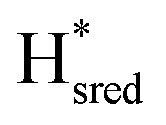
 transition has a more positive potential (−660 mV) than WT (−690 mV), and the H_ox_ to H_red_ and 
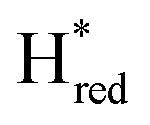
 to 
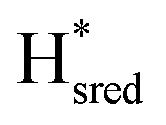
 transitions have more negative redox potentials (−480 mV and −690 mV, respectively) than WT (−440 mV and −670 mV, respectively). The oxidative titrations gave similar values of the potentials (Fig. S9†). The above observations suggest that mutation of S267 may have disrupted the redox-coupling between the two clusters and hence the activity of this variant is significantly lower than that of WT-*Tm*HydS. Furthermore, electron transfer from [2Fe]_H_ to [4Fe–4S]_H_ is less favorable in the S267M variant compared with the WT enzyme, explaining the lower activity and difficulty in oxidizing the variant. It should be noted that the redox potential for the 
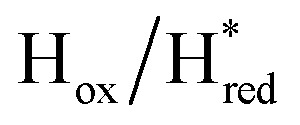
 transition in S267M^PDT^ (−450 mV) is 135 mV more negative than the same transition in S267M^ADT^ (−315 mV). A similar observation was also made for WT *Tm*HydS,^6^ and again agrees with the idea that [2Fe]^PDT^ has a more negative redox potential than [2Fe]^ADT^ in *Tm*HydS, and that this is due to the difference in electron withdrawing ability of the bridge-head atom of the dithiolate ligand.

## Discussion

In this work, we have explored the influence of amino acids surrounding the active site H-cluster in a recently discovered group of sensory [FeFe] hydrogenases, including the sensory hydrogenase HydS from *Thermotoga maritima*. These enzymes are strongly divergent from the well-characterized prototypical hydrogenases and, therefore, provide complementary information toward the understanding of the mechanism of [FeFe] hydrogenases in general. Furthermore, they provide insight on the important factors needed for functioning as efficient biological sensors.

We have investigated the function of the secondary coordination sphere amino acids by site-directed mutagenesis, generating seven *Tm*HydS variants. Our results show that changes to the protein environment have only subtle effects on the spectroscopic and catalytic properties in most cases. However, the serine residue present at position 267 plays an important role in *Tm*HydS, and appears to tune the potential of the H-cluster. We suggest that S267 influences the potential of [4Fe–4S]_H_ by stabilizing the reduced state of the cluster. This residue is not conserved in other sensory hydrogenases (HydS), so the question arises if the identity of this amino acid is indeed important for the function of sensory [FeFe] hydrogenases, and why it is not well-conserved? It is possible that the redox potential of the [4Fe–4S] cluster regulates the sensory properties of the enzyme and that changing the amino acid at the S267 position can fine-tune the sensory behaviour. A clue here is that oxidation of S267M was more difficult than for WT. This would be expected to make the S267M variant sensitive only at low concentrations of H_2_. For *Thermotoga maritima* to appropriately respond to changes in the H_2_ concentration a sensor is required that can respond to a wide range of H_2_ concentrations. It would be interesting to see whether the H_2_ concentrations found in various environments correlate with the amino acid at this position in HydS from other bacteria.

More generally, our work presents a conundrum: which amino acids form the proton transfer pathway in *Tm*HydS. Originally, we speculated that proton transfer in HydS may have been impeded due to the A131, which is normally occupied by cysteine in prototypical hydrogenases. However, the A131C variant actually had lower activity than WT. Firstly, this result supports the notion of an alternative proton transfer pathway, and, secondly, this suggests that interaction of cysteine with the ADT ligand in A131C HydS may alter the electronic structure of the H-cluster enough to interfere with catalysis. Another amino acid, Y223, located very close to the ADT ligand presents a possible starting point for an alternative proton transfer pathway. However, the Y223F variant lacking the hydroxyl group actually had increased H_2_ oxidation activity, in contrast to what would be expected for a crucial proton transfer pathway amino acid. S267 presented another possibility, and, at first, the extremely low activity of the S267M variant suggested a crucial role for this amino acid. However, the S267A variant retained 50% activity and so it seems unlikely that the hydroxyl group of S267 is a crucial proton transfer component. It cannot be excluded that a water molecule can substitute for the hydroxyl group, but it seems more likely that this amino acid does not constitute part of the proton transfer pathway.

The idea of alternative proton transfer pathways suggests that their evolution occurred quite late and that for a long time, primitive hydrogenase enzymes were functioning with only rudimentary proton transfer networks, possibly mediated by the diffusion of water to and from the active site. However, the selection pressure for fast and efficient H_2_ oxidation or proton reduction drove the evolution of highly tuned proton transfer pathways in modern prototypical [FeFe] hydrogenases. More research on diverse groups of [FeFe] hydrogenase is clearly needed to understand the evolution of the proton transfer pathway in more detail.

Overall, this work highlights the importance of tuning the properties of the two halves of the H-cluster, the [4Fe–4S] and [2Fe] subclusters, for enzyme function. In prototypical enzymes it appears to be crucial to maintain a very low redox potential for the [2Fe] subcluster so that it can only become reduced with concomitant protonation of the ADT ligand, ensuring efficient proton-coupled electron transfer. Furthermore, the redox potential for the [4Fe–4S] subcluster is close to the thermodynamic 2H^+^/H_2_ potential at pH 7 (*E* = −420 mV *vs.* SHE). In HydS the [2Fe] subcluster has quite a high redox potential and so it can be reduced without protonation of the ADT ligand. However, in HydS, the redox potential of the [4Fe–4S] cluster can be modulated to fine-tune the sensitivity of the H-cluster to H_2_.

## Conclusions

In this work, we explored the role of several amino acids surrounding the active site in the sensory [FeFe] hydrogenase, HydS, from *Thermotoga maritima*. Overall, only subtle effects were observed through mutagenesis of these amino acids, however, we identified one mutant with an altered redox potential of the [4Fe–4S]_H_ subcluster that led to a dramatic decrease in activity. These results highlight the importance of the secondary coordination sphere interactions in tuning active site properties, especially for specific functions such as sensing low concentrations of hydrogen.

## Data availability

The raw data has been uploaded to Zenodo and is available at DOI: 10.5281/zenodo.7737908.

## Author contributions

NC: conceptualization, methodology, software, data curation, writing – original draft preparation, visualization, investigation, writing – reviewing and editing. PR-M: methodology, software, data curation, writing – original draft preparation, investigation, writing – reviewing and editing. EJR: methodology, supervision, writing – reviewing and editing. WL: conceptualization, supervision, writing – reviewing and editing, funding acquisition. HO: conceptualization, supervision, writing – reviewing and editing, funding acquisition. JB: data curation, writing – original draft preparation, writing – reviewing and editing, funding acquisition.

## Conflicts of interest

There are no conflicts to declare.

## Supplementary Material

SC-014-D2SC06432D-s001
