# Supplementary material for: Redox tuning of the H-cluster by second coordination sphere amino acids in the sensory [FeFe] hydrogenase from Thermotoga maritima
Source: Chem Sci. 2023 Feb 27;14(13):3682–92. doi: 10.1039/d2sc06432d (PMC10055695; doi:10.1039/d2sc06432d)
Supplement: SC-014-D2SC06432D-s001 [file SC-014-D2SC06432D-s001.pdf]

Electronic Supplementary Material (ESI) for Chemical Science.
This journal is © The Royal Society of Chemistry 2023

Supporting information

Redox tuning of the H-cluster by second coordination sphere amino acids in the sensory [FeFe] hydrogenase from *Thermotoga maritima*

Nipa Chongdar,*[a,b] Patricia Rodríguez-Maciá [a,c], Edward J. Reijerse[a], Wolfgang Lubitz[a], Hideaki Ogata*[d] and James A. Birrell*[a,e]

[a.] Max Planck Institute for Chemical Energy Conversion, Stiftstraße 34-36, 45470 Mülheim an der Ruhr, Germany

[b.] CSIR-National Institute of Oceanography, Dona Paula - 403004, Goa, India

[c.] Department of Chemistry, Inorganic Chemistry Laboratory, University of Oxford, South Parks Road, Oxford, OX1 3QR, UK

[d.] Graduate School of Life Science, University of Hyogo, Koto 3-2-1, Kamigori, Ako, 678-1297 Hyogo, Japan

[e.] School of Life Sciences, University of Essex, Colchester, CO4 3SQ, UK

Experimental section

Construction of *Tm*HydS variants

The point mutations at positions A131, G177, Y223 and S267 were introduced using a site directed mutagenesis protocol, but with partially overlapping primers (listed in table S1) and plasmid pASK-IBA3plus-*Tm*HydS[1] as the template. Introduction of the mutations in the resultant plasmids was confirmed using DNA sequencing.

Protein expression and purification

The plasmid DNAs of *Tm*HydS wildtype (WT) and its variants were transformed into *Escherichia coli* BL21 (DE3) *ΔiscR* cells[2] and single colonies were grown at 37 °C in LB-agar media supplemented with 100 µg ml^{-1} ampicillin and 50 µg ml^{-1} kanamycin. Saturated overnight cultures grown from these single colonies were then used to inoculate LB broth (supplemented with antibiotics: ampicillin, kanamycin, 2 mM ferric ammonium citrate, 2 mM L-cysteine, 0.5% (w/v) glucose and 100 mM MOPS/NaOH to make the final pH of the medium 7.4) for protein expression. These cultures were grown aerobically at 37 °C to an OD_{600} of ≈ 0.4 – 0.6. These cultures were then supplemented with 2 mM L-cysteine and protein expression was induced by addition of 200 µg ml^{-1} anhydrotetracycline. Cultures were transferred to glass bottles, sealed with lids containing PTFE/silicone septa, sparged with argon gas for 2 h, and incubated for ≈ 20 h at room temperature. The cells were then harvested anaerobically at 4000 x g by centrifugation and were stored at -80 °C until further use.

All the following steps for protein purification were performed under strictly anaerobic conditions in an anaerobic chamber containing 1.5 – 2% H_2. All glass and plastic-ware, as well as the buffers used for the experiments, were incubated inside the chamber for at least 24 h before use. Cells were resuspended in lysis buffer containing 0.1 M Tris-HCl pH 8, 0.15 M NaCl, 2 mM sodium dithionite and EDTA free protease inhibitor cocktail (Roche). The cell suspension was disrupted by sonication on ice and the crude lysate was clarified by centrifugation (45000 x g) followed by filtration through a 0.44 µm membrane filter (Millipore). WT-*Tm*HydS and its variants were purified on a 10 ml Streptactin superflow resin pre-equilibrated in buffer (0.1 M Tris-HCl pH 8, 0.15 M NaCl, 2 mM sodium dithionite). After loading, the column was washed with the same buffer and the protein was eluted with the same buffer containing 2.5 mM d-desthiobiotin. The proteins were then concentrated to ≈ 0.5 – 0.7 mM and stored at – 80 °C until further use. The protein concentrations were measured using Bio-Rad DC Protein Assay Reagent using BSA (Bovine Serum Albumin) as a standard. Protein iron determination was performed using the method published by Huberman and Perez.[3] The samples were judged to be pure by SDS-PAGE analysis.

Artificial maturation of *Tm*HydS variants with $(Et_4N)_2[Fe_2(ADT)(CO)_4(CN)_2]$ and $(Et_4N)_2[Fe_2(PDT)(CO)_4(CN)_2]$

$(Et_4N)_2[Fe_2(ADT)(CO)_4(CN)_2]$ and $(Et_4N)_2[Fe_2(PDT)(CO)_4(CN)_2]$ were synthesised as described previously.[4-5] The compounds were dissolved in dry dimethyl sulfoxide (DMSO) and stored at – 80 °C. The procedure of artificial maturation of the *Tm*HydS variants was identical to what was reported previously for the WT-*Tm*HydS.[1] The artificially maturated proteins were concentrated to 0.5 – 1 mM using Amicon Ultracentrifugal filters (50 kDa) and stored at – 80 °C. Before measurement of FTIR and EPR spectroscopy, the holo-proteins were exchanged into 50 mM Tris-HCl pH 8, 200 mM KCl buffer.

Activity assays

Hydrogen production was measured by gas chromatography on a 6890 Series GC System (Agilent Technologies) using a molecular sieve 5 Å PLOT column using reduced methyl viologen as the electron donor. The 400 µl reactions were set up in 2.5 mL stoppered plastic tubes containing 10 µg – 50 µg of artificially maturated *Tm*HydS variants in 200 mM potassium phosphate buffer pH 8, 100 mM sodium dithionite, and 10 mM methyl viologen. The reactions were carried out at 70 °C and they were initiated by addition of the protein. The reaction vials were purged with argon for 5 min and incubated in a temperature-controlled water bath at 70 °C for 10 min before extraction of 0.3 mL of the headspace gas for analysis. Hydrogen content was quantified by comparison with a 100% H_2 standard. All values are the average of three measurements after subtracting the value of a blank measurement.

Hydrogen oxidation was measured by following the reduction of 1 mM benzyl viologen in H_2 – saturated 200 mM phosphate buffer pH 8 with 1.0 µg – 10 µg artificially maturated *Tm*HydS variants. The specific activity of the protein was measured by the initial rate of change of absorbance at 600 nm due to the reduction of benzyl viologen. The measurements were performed in 1.5 ml plastic cuvettes using an Ocean Optics DH-mini UV-Vis-NIR light source and a USB2000 + XR1-ES detector, operated by the SpectraSuite software. The reactions were carried out at 70 °C using a temperature controlled cuvette holder (CUV-QPOD-2E-ABSKIT, Ocean Optics). All values are the average of three measurements after subtracting the value of the blank measurement. The other details are described in the figure legends.

EPR spectroscopy

For X-band (9.63 GHz) EPR spectroscopy, 200 µl of samples were transferred to X-band quartz EPR tubes and frozen in liquid nitrogen. The spectra were recorded on a Bruker ELEXSYS E500 CW EPR spectrometer. Cryogenic temperatures were maintained with liquid He using an Oxford ESR900 helium flow cryostat. The measurement parameters were: modulation frequency, 100 kHz, modulation amplitude, 7.46 G, time constant, 81.92 ms, conversion time, 81.92 ms. All the spectra were processed using home written programs in the MATLAB™ environment. EPR simulations were also carried out in MATLAB™ using the 'esfit' fitting function from the Easyspin package.[6]

FTIR spectroscopy and spectroelectrochemistry

FTIR spectra of the samples were recorded using a Bruker IFS 66v/S FTIR spectrometer equipped with a liquid nitrogen cooled Bruker mercury cadmium telluride (MCT) detector. For FTIR spectroscopy, 10 µl of sample were placed between two CaF_2 windows (Korth Kristalle, Altenholz), separated by a 50 µm Teflon spacer. These windows were then accommodated in a FTIR cell and fixed with rubber rings. Spectra were collected at 15 °C in the double sided, forward-backward mode with 1000 scans, and a resolution of 2 cm^{-1}, an aperture setting of 2 mm and scan velocity of 20 kHz.

Spectroelectrochemical FTIR was carried out in the same spectrometer set-up, but using a home built electrochemical IR cell, constructed according to an original design by Moss et. al.[7] Protein samples (≈ 1 - 1.5 mM, 30 µl) mixed with 0.5 mM of each redox mediator (potassium indigo trisulphonate, anthraquinone-1,5-disulfonic acid, anthraquinone-2-sulfonate, benzyl viologen, methyl viologen and 1,1´,2,2´-tetramethyl-[4,4´-bipyridine]-1,1´-diium iodide) and loaded between two CaF_2 windows on an electrochemically reduced gold mesh working electrode (approximately 50 µm thick) in electrical contact with a platinum counter electrode. An Ag/AgCl (1 M KCl) electrode was used as a reference and was calibrated before and after measurement with (hydroxymethyl)ferrocine (E = + 436 mV vs SHE). The potential was set using an Autolab PGSTAT101 potentiostat controlled by Nova software. The sample was equilibrated at a particular potential until the current flowing through the cell reached an equilibrium plateau, which took ≈ 30 – 75 min. This was followed by measurement of the FTIR spectrum at that potential. All the FTIR spectra were processed using home written routines in the MATLAB™ environment.

Table S1. Sequences of the primers used for mutagenesis

Primer name	Primer sequence
A131C_for	5’- ACAACG***TGC***TGCCCTGTGGTTGTGAATC-3’
A131C_rev	5’-AGGGCA***GCA***CGTTGTTATGAAGGGAC-3’
G177M_for	5’-TGTGTTTGTT***AGC***CCATGCATAGCAAAGAAATCC-3’
G177M_rev	5’-TATGCATGG***GCT***AACAAACACAATTGGGAAGTCAC -3’
Y223F_for	5’-GCTCGGTTT***TTC***CCGACAACAGATGGTATAGGC-3’
Y223F_rev	5’-GTTGTCGG***GAA***AAACCGAGCCCTGTCTGGATACG-3’
S267M_for	5’-CATAGAGGCT***ATG***GCATGCTACGGCAGCTGTTTG-3’
S267M_rev	5’-GCCGTAGCATGC***CAT***AGCCTCTATGAAAACACTTTTG-3’
S267A_for	5’-CATAGAGGCT***GCG***GCATGCTACGGCAGCTGTTTG-3’
S267A_rev	5’-GCCGTAGCATGC***CGC***AGCCTCTATGAAAACACTTTTG-3’
S267C_for	5’-CATAGAGGCT***TGC***GCATGCTACGGCAGCTGTTTG-3’
S267C_for	5’-GCCGTAGCATGC***GCA***AGCCTCTATGAAAACACTTTTG-3’
S267D_for	5’-CATAGAGGCT***GAT***GCATGCTACGGCAGCTGTTTG-3’
S267D_rev	5’-GCCGTAGCATGC***ATC***AGCCTCTATGAAAACACTTTTG-3’

Table S2. Iron content of apo WT-*Tm*HydS and its variants

**Protein**	**Fe (per mole of protein)**
WT	14.7 ± 1.1
A131C	14.4 ± 1.2
G177M	13.9 ± 0.7
Y223F	16.0 ± 0.9
S267M	14.3 ± 1.7

Supplementary Figures

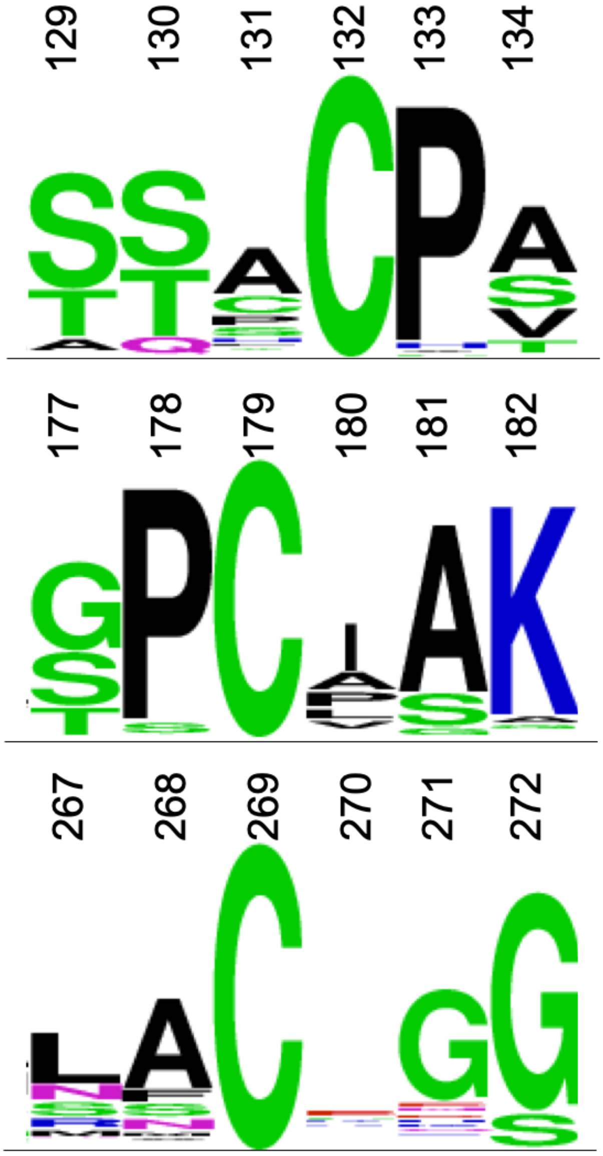

Figure S1. WebLogo plot (prepared using 251 sequences from the HydDB server[8]) depicting the conservation pattern of amino acids mutated in this study and their vicinity in sensory hydrogenases. The overall height of each stack of letters in the logo plot indicates sequence conservation at a particular position, and the height of each amino acid one-letter code in each stack shows the frequency of occurrence of those amino acids in that position.

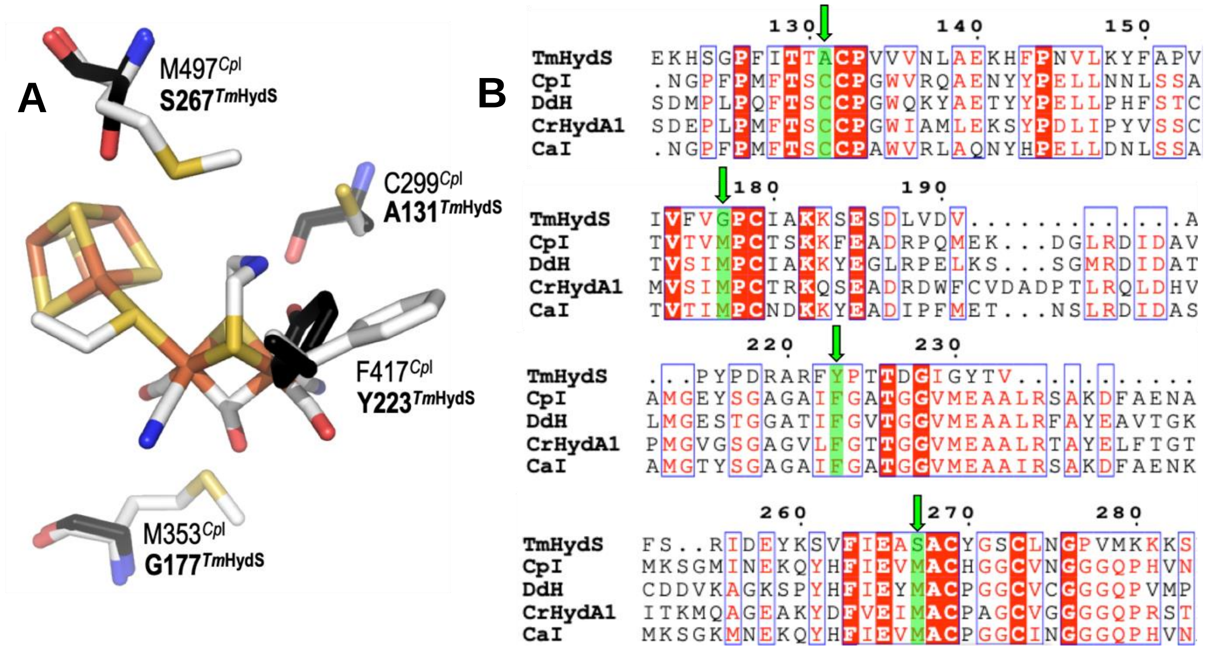

Figure S2. A) The CpI structure (4XDC)[9] and the homology model of *Tm*HydS (built in the SWISS-MODEL server using the crystal structure of *Dd*HydAB (1HFE)[10] as a template) are shown in gray and black, respectively. The homology model reveals the presence of a tyrosine residue (Y223) in the vicinity of the ADT bridge in *Tm*HydS, which is replaced by the phenylalanine (F417) in *Cp*I. B) Partial sequence alignment of *Tm*HydS with prototypical hydrogenases (*Cp*I - *Clostridium pasteurianum*, *Ca*I - *Clostridium acetobutylicum*, *Dd*H – *Desulfovibrio desulfuricans*, *Cr*HydA1 - *Chlamydomonas reinhardtii*) showing the differences in identities of amino acids in *Tm*HydS compared to prototypical hydrogenases at positions 131, 177, 223, and 267 (marked with green arrows).

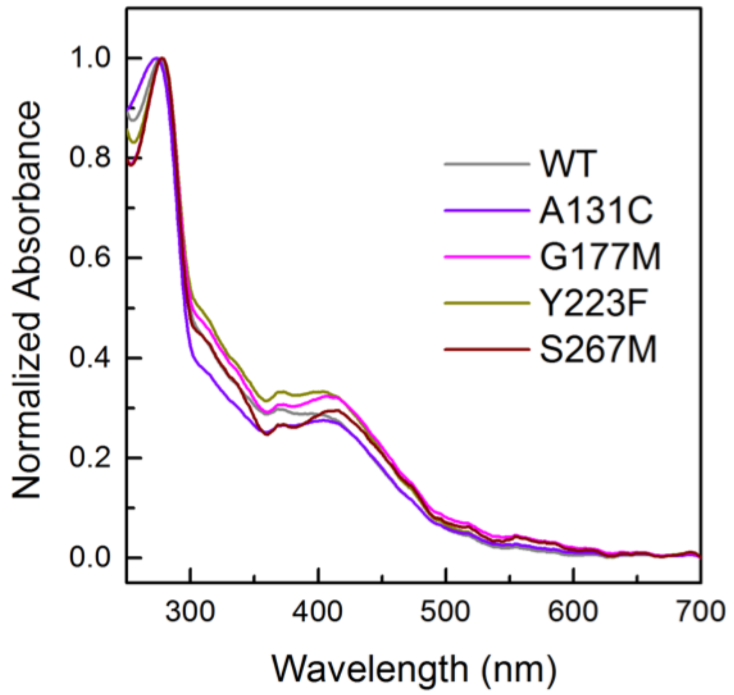

Figure S3. UV-vis spectra of apo WT-*Tm*HydS and its variants in 0.1 M Tris-HCl pH 8 and 0.15 M NaCl.

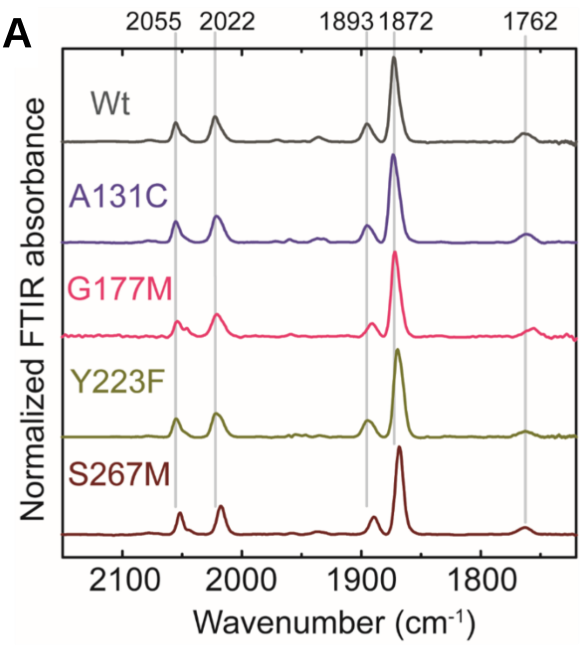

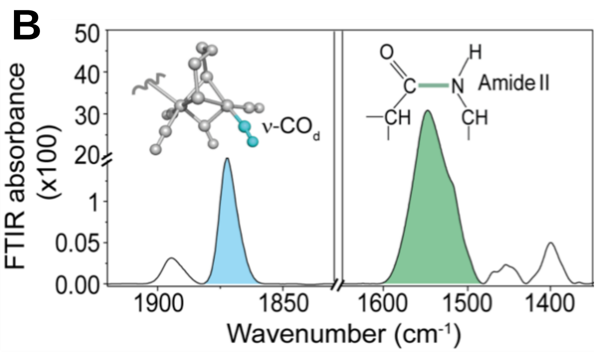

C

Protein	Ratio (x 10^{-3})
WT	8.0 ± 1.5
A131C	6.5 ± 1.5
G177M	4.0 ± 1.0
Y223F	10.5 ± 1.0
S267M	7.5 ± 1.5

Figure S4. A) The FTIR spectra of holo-WT-*Tm*HydS and its variants at 15 °C in the as-isolated form under 2% H_2 /98% N_2 at pH 8. B) FTIR absorbance bands of the most intense CO stretching band (≈ 1872 cm^{-1}) and the amide II band (≈ 1540 cm^{-1}) are shown. C) The ratio between the intensities of the bands at ≈ 1872 cm^{-1} and ≈ 1540 cm^{-1} are tabulated.

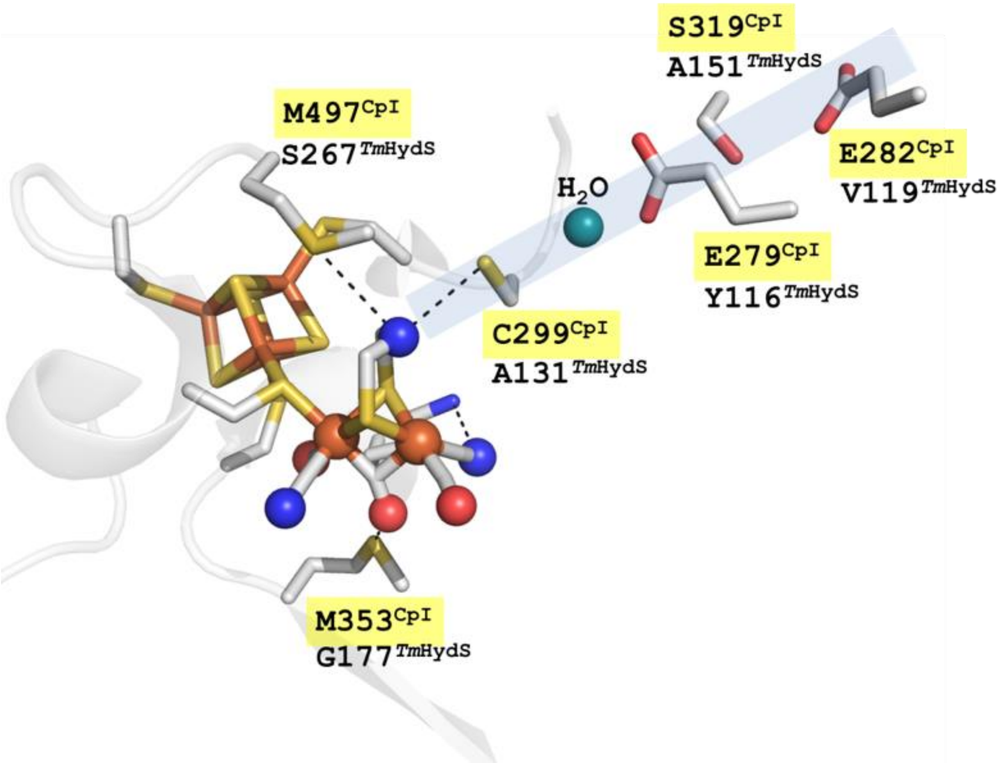

Figure S5. Magnified view of the H-cluster and the proton channel in the *Cp*I hydrogenase.[9] The water molecule involved in proton transfer is shown as a sphere (turquoise). All the proton channel residues, C299, S319, E279 and E282 are altered in *Tm*HydS.

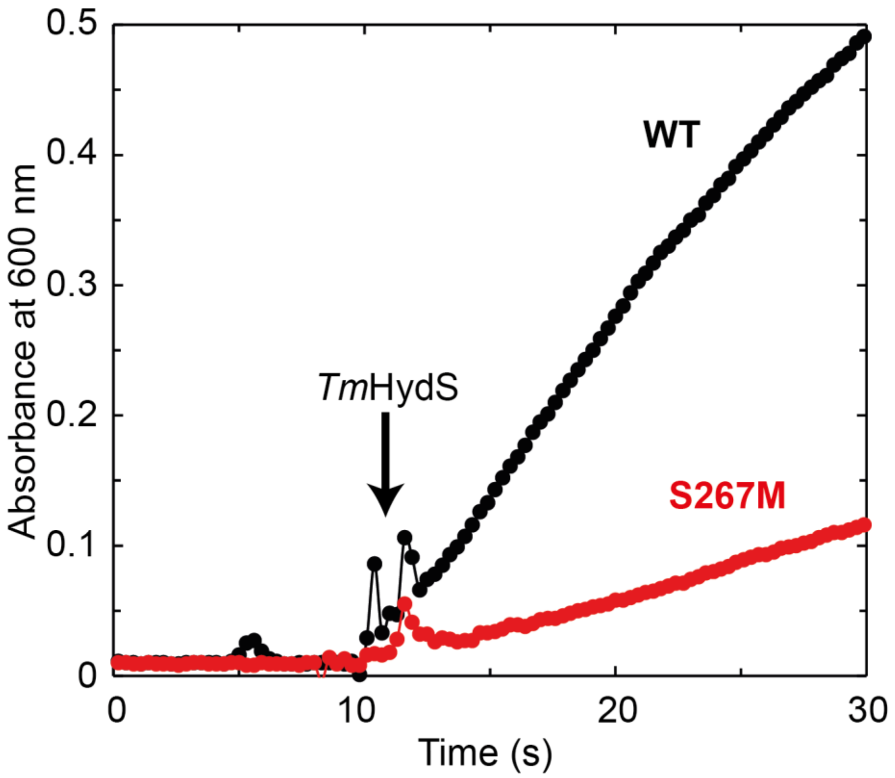

Figure S6. Kinetic traces of H_2-dependent reduction of 1 mM benzyl viologen monitored by absorbance at 600 nm for WT *Tm*HydS (black trace) and the S267M variant (red trace) at 70 ^{o}C in 200 mM potassium phosphate, pH 8. At approximately 10 s the reaction was initiated by addition of enzyme (0.9 µg of WT or 2.5 µg of S267M).

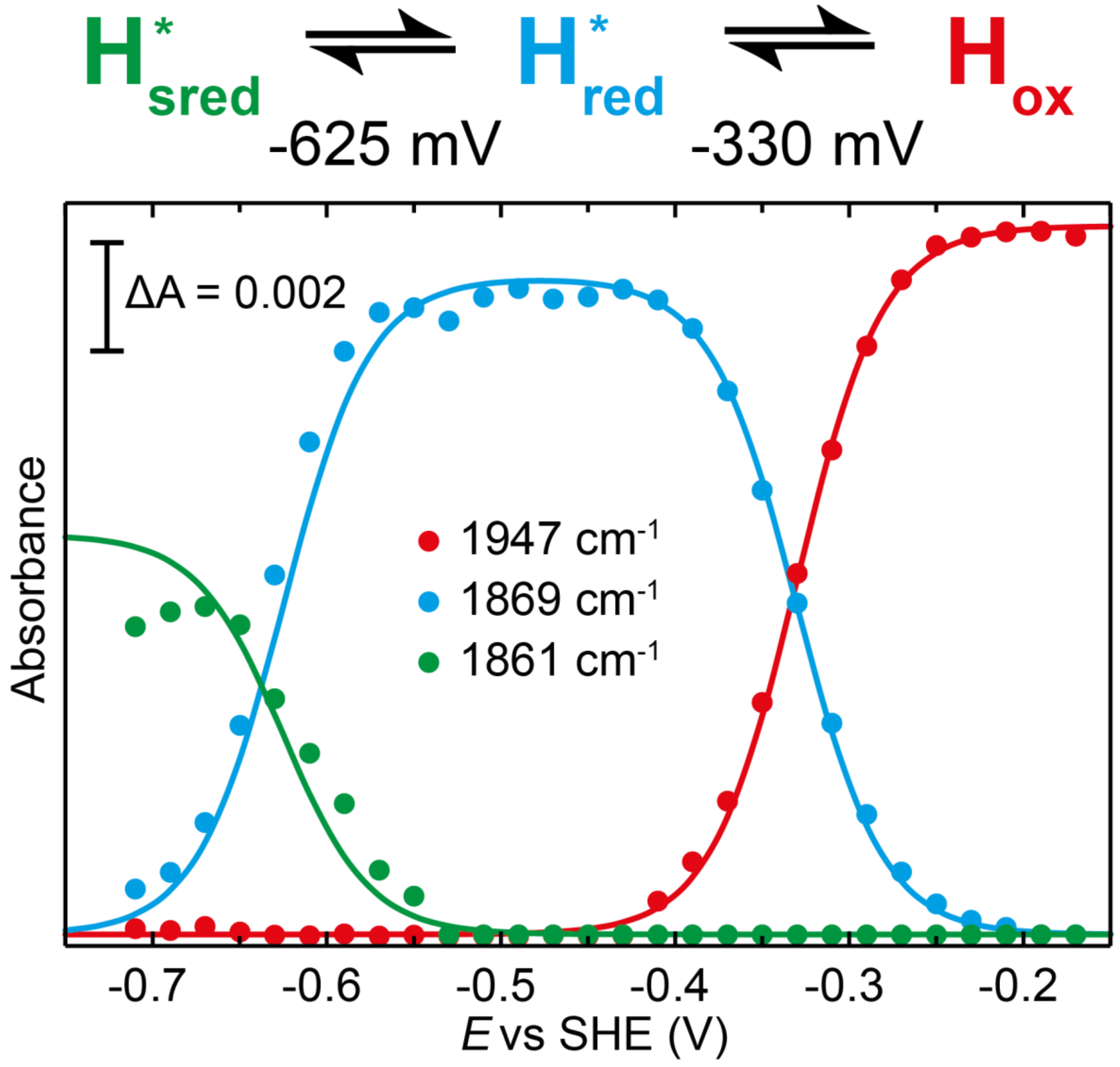

Figure S7. Oxidative IR spectroelectrochemical titration of *Tm*HydS^{ADT} S267M. The intensity of the most intense band in the H_{ox} (1947 cm^{-1}), H^{*}_{red} (1869 cm^{-1}) and H^{*}_{sred} (1861 cm^{-1}) states, the latter two were determined from fitting Gaussian peaks (see Figure S8), is plotted against the applied potential and fitted with a model based on the Nernst equation using n = 1 for the H_{ox}/H^{*}_{red} (E_m = –330 mV) and H^{*}_{red} /H^{*}_{sred} (E_m = –625 mV) transitions. These data represent the reverse titration (re-oxidation) of the IR spectroelectrochemical titration presented in Fig. 6 of the main text.

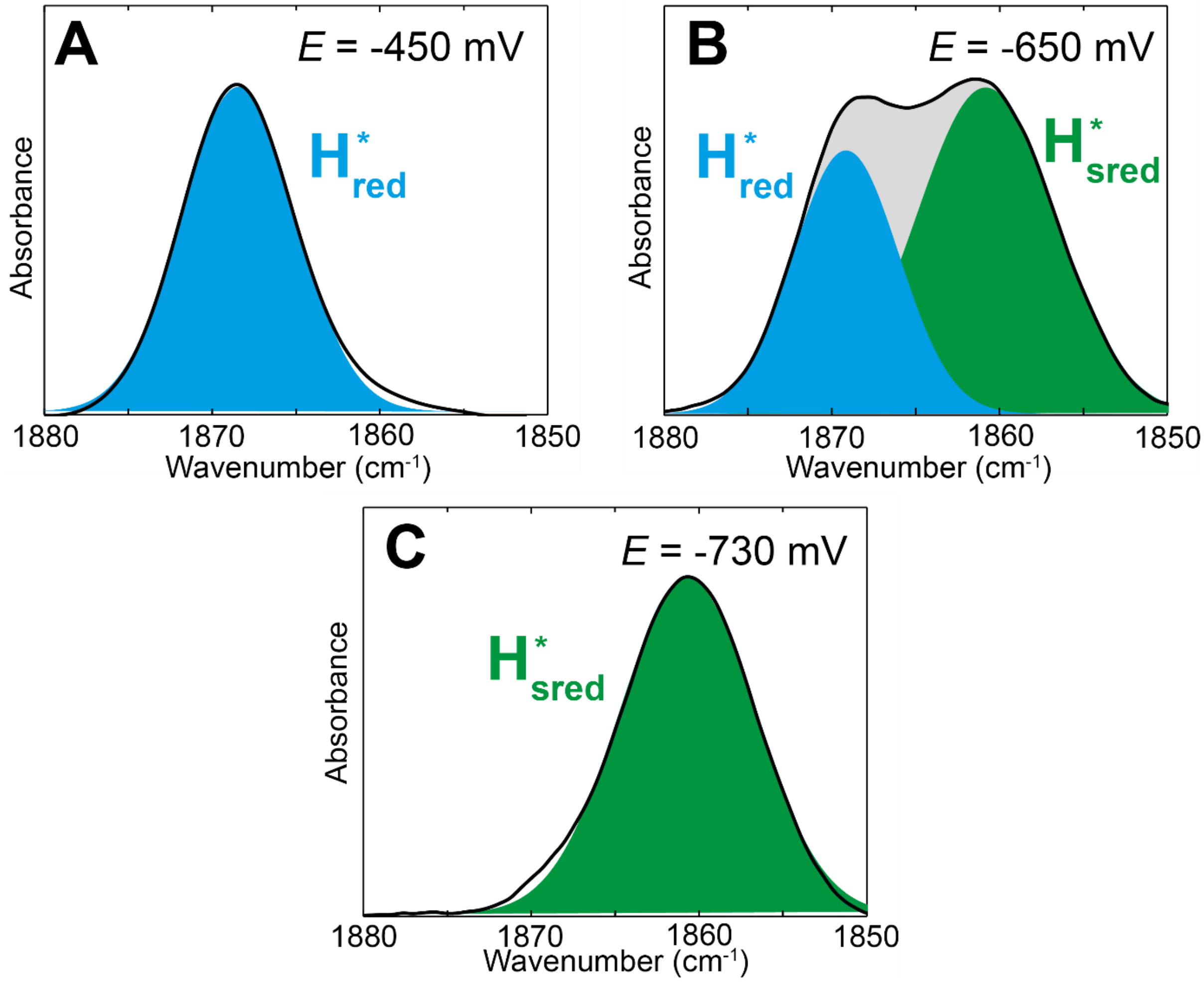

Figure S8. Gaussian peak fitting of the most intense peak in the H^{*}_{red} (blue) and H^{*}_{sred} (green) states of *Tm*HydS^{ADT} S267M to obtain the titration curves presented in Figures 6 and S7. The 1850 cm^{-1} to 1880 cm^{-1} region of the IR spectra at three exemplary applied potentials during the reductive titration are shown where there is pure H^{*}_{red} (**A**, *E* = –450 mV), a mixture of H^{*}_{red} and H^{*}_{sred} (**B**, *E* = –650 mV), and pure H^{*}_{sred} (**C**, *E* = –730 mV).

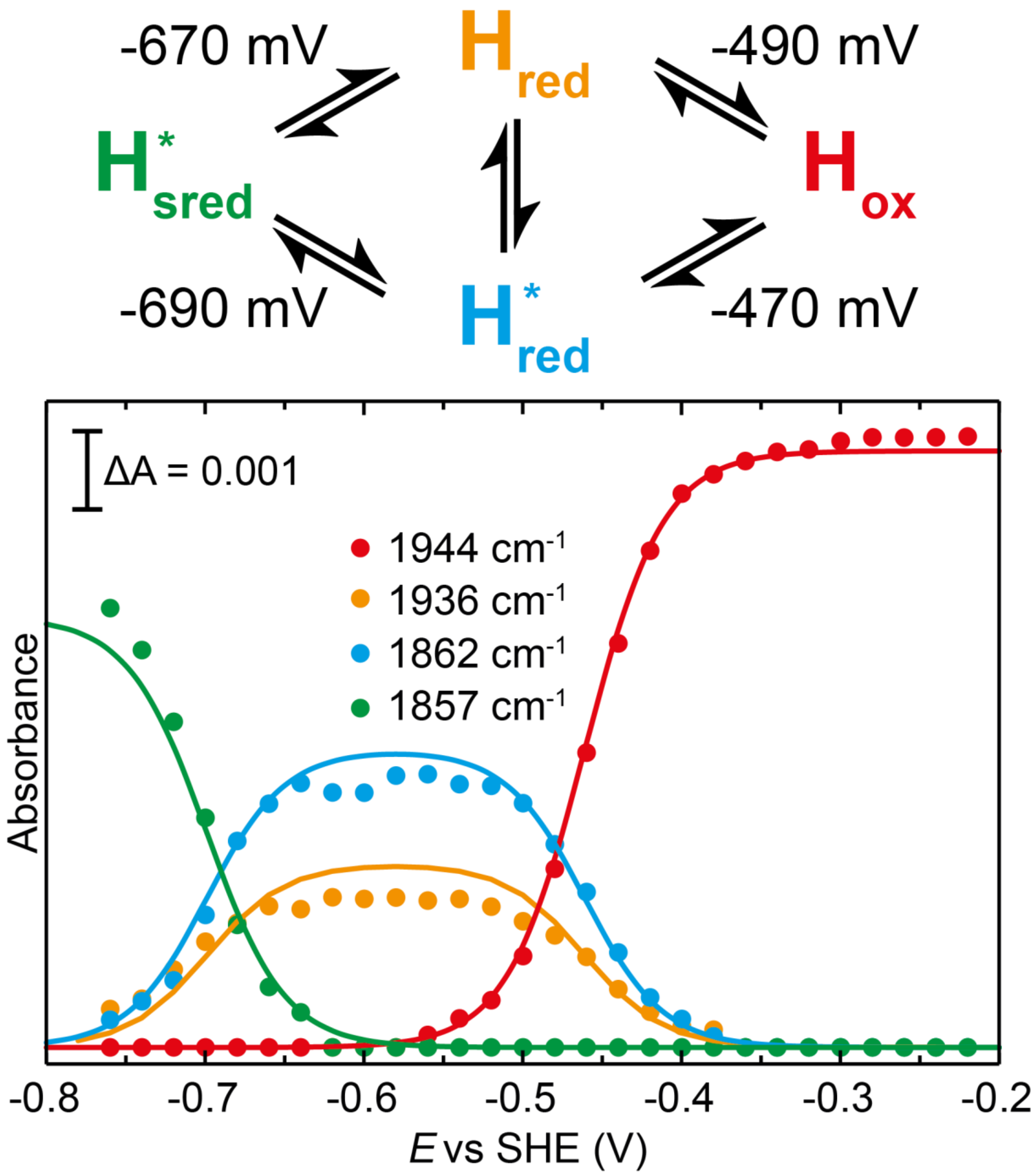

Figure S9. Oxidative IR spectroelectrochemical titration of *Tm*HydS^{PDT} S267M. The intensity of the most intense band in the H_{ox} (1944 cm^{-1}), H_{red} (1936 cm^{-1}), H^{*}_{red} (1862 cm^{-1}) and H^{*}_{sred} (1857 cm^{-1}) states, all determined from fitting Gaussian peaks (see Figure S10), is plotted against the applied potential and the data are fitted with a model based on the Nernst equation using n = 1 for the H_{ox}/H_{red} (E_m = –490 mV), H_{ox}/H^{*}_{red} (E_m = –460 mV), H_{red}/H^{*}_{sred} (E_m = –670 mV) and H^{*}_{red}/H^{*}_{sred} (E_m = –690 mV) transitions. These data represent the reverse titration (re-oxidation) of the IR spectroelectrochemical titration presented in Fig. 7 of the main text.

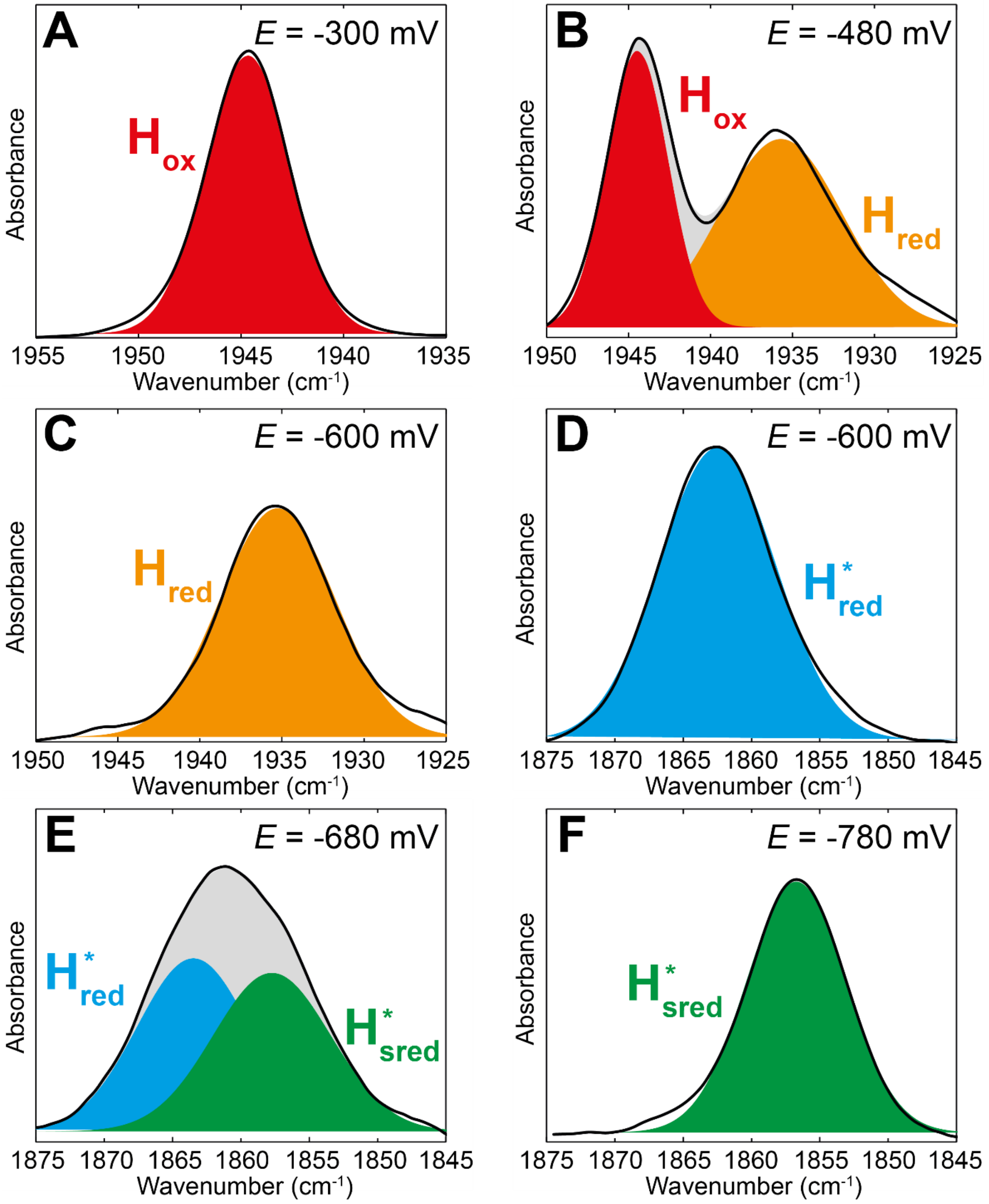

Figure S10. Gaussian peak fitting of the most intense peak in the H_{ox} (red), H_{red} (orange), H^*_{red} (blue) and H^*_{sred} (green) states of *Tm*HydS^{PDT} S267M to obtain the titration curves presented in Figures 7 and S9. The 1925 cm^{-1} to 1955 cm^{-1} (**A – C**) and 1845 cm^{-1} to 1875 cm^{-1} (**D – F**) regions of the IR spectra at five exemplary applied potentials during the reductive titration are shown where there is pure H_{ox} (**A**, *E* = –300 mV), a mixture of H_{ox} and H_{red} (**B**, *E* = –480 mV), pure H_{red} (**C**, *E* = –600 mV), pure H^*_{red} (**D**, *E* = –600 mV), a mixture of H^*_{red} and H^*_{sred} (**E**, *E* = –680 mV), and pure H^*_{sred} (**C**, *E* = –780 mV).

Supplementary references

1. Chongdar, N.; Birrell, J. A.; Pawlak, K.; Sommer, C.; Reijerse, E. J.; Rüdiger, O.; Lubitz, W.; Ogata, H., Unique spectroscopic properties of the H-cluster in a putative sensory [FeFe] hydrogenase. *J. Am. Chem. Soc.* **2018,** *140* (3), 1057-1068.
2. Kuchenreuther, J. M.; Grady-Smith, C. S.; Bingham, A. S.; George, S. J.; Cramer, S. P.; Swartz, J. R., High-yield expression of heterologous [FeFe] hydrogenases in *Escherichia coli*. *PLoS One* **2010,** *5* (11), e15491.
3. Huberman, A.; Pérez, C., Nonheme iron determination. *Anal. Biochem.* **2002,** *307* (2), 375-378.
4. Li, H.; Rauchfuss, T. B., Iron carbonyl sulfides, formaldehyde, and amines condense to give the proposed azadithiolate cofactor of the Fe-only hydrogenases. *J. Am. Chem. Soc.* **2002,** *124* (5), 726-727.
5. Le Cloirec, A.; C. Davies, S.; J. Evans, D.; L. Hughes, D.; J. Pickett, C.; P. Best, S.; Borg, S., A di-iron dithiolate possessing structural elements of the carbonyl/cyanide sub-site of the H-centre of Fe-only hydrogenase. *Chem. Commun.* **1999,** (22), 2285-2286.
6. Stoll, S.; Schweiger, A., EasySpin, a comprehensive software package for spectral simulation and analysis in EPR. *J. Mag. Res.* **2006,** *178* (1), 42-55.
7. Moss, D.; Nabedryk, E.; Breton, J.; Mäntele, W., Redox-linked conformational changes in proteins detected by a combination of infrared spectroscopy and protein electrochemistry. *Eur. J. Biochem.* **1990,** *187* (3), 565-572.
8. Søndergaard, D.; Pedersen, C. N. S.; Greening, C., HydDB: A web tool for hydrogenase classification and analysis. *Sci. Rep.* **2016,** *6* (1), 34212.
9. Esselborn, J.; Muraki, N.; Klein, K.; Engelbrecht, V.; Metzler-Nolte, N.; Apfel, U. P.; Hofmann, E.; Kurisu, G.; Happe, T., A structural view of synthetic cofactor integration into [FeFe]-hydrogenases. *Chem. Sci.* **2016,** *7* (2), 959-968.
10. Nicolet, Y.; Piras, C.; Legrand, P.; Hatchikian, C. E.; Fontecilla-Camps, J. C., *Desulfovibrio desulfuricans* iron hydrogenase: the structure shows unusual coordination to an active site Fe binuclear center. *Structure* **1999,** *7* (1), 13-23.
